# Cross-species transmission of a novel bisegmented orfanplasmovirus in the phytopathogenic fungus *Exserohilum rostratum*

**DOI:** 10.3389/fmicb.2024.1409677

**Published:** 2024-05-23

**Authors:** Jichun Jia, Linjie Nan, Zehao Song, Xu Chen, Jinsheng Xia, Lihong Cheng, Baojun Zhang, Fan Mu

**Affiliations:** ^1^College of Plant Protection, Shanxi Agricultural University, Jinzhong, Shanxi, China; ^2^Shanxi Key Laboratory of Integrated Pest Management in Agriculture, Taiyuan, Shanxi, China

**Keywords:** mycovirus, *Exserohilum rostratum*, orfanplasmovirus, vertically transmission, cross-species transmission

## Abstract

Mycoviruses have been found in various fungal species across different taxonomic groups, while no viruses have been reported yet in the fungus *Exserohilum rostratum.* In this study, a novel orfanplasmovirus, namely Exserohilum rostratum orfanplasmovirus 1 (ErOrfV1), was identified in the *Exserohilum rostratum* strain JZ1 from maize leaf. The complete genome of ErOrfV1 consists of two positive single-stranded RNA segments, encoding an RNA-dependent RNA polymerase and a hypothetical protein with unknown function, respectively. Phylogenetic analysis revealed that ErOrfV1 clusters with other orfanplasmoviruses, forming a distinct phyletic clade. A new family, Orfanplasmoviridae, is proposed to encompass this newly discovered ErOrfV1 and its associated orfanplasmoviruses. ErOrfV1 exhibits effective vertical transmission through conidia, as evidenced by its 100% presence in over 200 single conidium isolates. Moreover, it can be horizontally transmitted to *Exserohilum turcicum*. Additionally, the infection of ErOrfV1 is cryptic in *E. turcicum* because there were no significant differences in mycelial growth rate and colony morphology between ErOrfV1-infected and ErOrfV1-free strains. This study represents the inaugural report of a mycovirus in *E. rostratum*, as well as the first documentation of the biological and transmission characteristics of orfanplasmovirus. These discoveries significantly contribute to our understanding of orfanplasmovirus.

## 1 Introduction

Mycoviruses are viruses that replicate in fungi or oomycetes and have been discovered across all major fungal groups (Xie and Jiang, 2014; Ghabrial et al., 2015; Sato and Suzuki, 2023). Recent metagenomic and metatranscriptomic analyses have significantly expanded the repertoire of mycoviruses, resulting in the identification of numerous novel taxa such as *Fusaviridae*, *Alternaviridae,* and proposed Splipalmiviridae (Sutela et al., 2020; He et al., 2022; Kondo et al., 2022; Ye et al., 2023; Zhang et al., 2023; Li et al., 2024). Most mycoviruses establish latent infections with no discernible impact on their fungal host. However, some mycoviruses can cause morphological alterations in their fungal hosts, such as hypovirulence (reduction of pathogenicity) or hypervirulence (significant increase in virulence) (Xie and Jiang, 2014; Kotta-Loizou and Coutts, 2017; Das et al., 2023; Wang et al., 2023). The mycoviruses associated with hypovirulence have the potential to control fungal diseases and offer valuable resources for investigating fungal biological mechanisms (Ghabrial and Suzuki, 2009; Kondo et al., 2022; Pedersen and Marzano, 2023; Hillman and Turina, 2024; Zhu et al., 2024).

With the advancement of high-throughput sequencing technologies, the fungal (+)ssRNA virus groups within the phylum *Lenarviricota* have experienced significant expansion, including members of the viral families *Narnaviridae*, *Mitoviridae,* and *Botourmiaviridae*, alongside numerous unclassified related viruses (Kondo et al., 2022). Members in the family *Narnaviridae* contain a non-segmented positive single-stranded RNA genome ranging from 2.3 to 2.9 kb, which encode a protein with characteristics of an RNA-dependent RNA polymerase (RdRp), including *Saccharomyces cerevisiae* 20S and 23S narnaviruses (ScNV20S and ScNV23S) (Hillman and Cai, 2013). The identification of numerous narna-like viruses, including splipalmiviruses, ambinarnaviruses and other unclassed narnaviruses, has expanded the understanding of this viral family (Kondo et al., 2022). Splipalmiviruses, characterized by a split polymerase palm domain and 2–4 genome segments, have been identified in diverse fungi, representing a novel taxonomic group within the narnaviruses (Sutela et al., 2020; Chiba et al., 2021; Ruiz-Padilla et al., 2021). Ambinarnaviruses represent a unique group of narna-like viruses known for their ambisense RNA genomes. Their genomes possess a reverse open reading frame (ORF) that spans nearly the full length of the negative-strand of the viral genome, in addition to a large ORF in the positive-strand (Retallack et al., 2021). Additionally, bisegmented orfanplasmoviruses have been reported in various fungi, suggesting their widespread distribution and potential relationship to narna-like viruses (Chiapello et al., 2020; Jia et al., 2021; Huang et al., 2023). To date, 15 orfanplasmoviruses have been reported, while only three viruses, namely Sclerotinia sclerotiorum narnavirus 3 (SsNV3), Sclerotinia sclerotiorum narnavirus 4 (SsNV4), and Fusarium asiaticum narnavirus 1 (FaNV1), were identified with the full-length sequence (Chiapello et al., 2020; Jia et al., 2021; Huang et al., 2023). However, the impact of orfanplasmoviruses on their host and their transmission characteristics remain unexplored.

Previous studies have suggested that mycoviruses have a narrow host range due to constraints on their horizontal transmission caused by vegetative incompatibility (Ghabrial and Suzuki, 2009). However, as metatranscriptomic research advances, scholars have found that this may not always be the case. Some highly similar mycoviruses have been detected in different fungal hosts, even those distantly related in taxonomy. For instance, a RNA virus from *Sclerotinia homoeocarpa* shares 92.4% nucleotide and 95.1% amino acid sequence identities with the Ophiostoma novo-ulmi mitovirus 3a-Ld from *Ophiostoma novo-ulmi* (Deng et al., 2003). Increasing evidence suggests that mycoviruses may have a broader host range in nature and could potentially spread between different hosts through unknown mechanisms (Khalifa and MacDiarmid, 2019; Song et al., 2022). Additionally, some studies indicate that under laboratory conditions, mycoviruses can achieve cross-species transmission in certain fungi (Melzer et al., 2002; Ning et al., 2022). Recently, a mycovirus was discovered that can cross-class transmit between *Leptosphaeria biglobosa* and *Botrytis cinerea* in nature (Deng et al., 2022). Even more surprisingly, a virus was identified from the phytopathogenic fungus (*Valsa mali*) of Valsa canker infecting apple orchards, which can be naturally spread to both apple plants and *V. mali* (Dai et al., 2024).

*Exserohilum rostratum*, a widespread phytopathogenic fungus, can infect more than 30 plant species, including economically significant crops such as corn, wheat, and rice (Cardona and González, 2007; Lin et al., 2011; Xie et al., 2022; Korra et al., 2023). Leaf spot disease induced by *E. rostratum* can lead to severe yield losses. For instance, pineapple leaf spot caused by *E. rostratum* eventually resulted in a 3–10% reduction of yield in the Hainan Province of China (Luo et al., 2012). This pathogen is also associated with corn leaf spot, affecting 18% of corn foliage and a considerable 85% of corn seeds with infection (Leonard et al., 1988). Furthermore, *E. rostratum* is capable of infecting humans and animals, causing meningitis, rhinosinusitis, keratitis, etc. (Litvintseva et al., 2014; Bennett et al., 2019). Despite the ubiquity of mycoviruses in fungi, no mycovirus infecting *E. rostratum* has been reported to date. In this study, we present the molecular and biological characteristics of a novel orfanplasmovirus from *E. rostratum,* representing the first report of a mycovirus infecting this fungus.

## 2 Materials and methods

### 2.1 Fungal isolate, growth conditions, and assays of biological properties

The strain JZ1 was isolated from a diseased maize leaf collected in Jinzhong, Shanxi province, China, in 2018. The diseased leaf was sterilized with 75% ethanol for 1 min and then rinsed three times with sterilized distilled water. Portions of the leaf were placed on potato dextrose agar (PDA) medium and cultivated at 25°C for 5 days. Subsequently, mycelial agar plugs were transferred to fresh PDA plates to obtain the strain JZ1. The strain JZ1 was stored on PDA slants or in 20% glycerol water at 4°C. The *E. turcicum* strain CZ2 and 5–1-1, *E. rostratum* strain 193 and *Alternaria alternata* strain were cultivated at 25°C and stored on PDA slants or in 20% glycerol water at 4°C.

A 6 mm mycelial plug of the strains was inoculated onto the center of a 9 cm petri dish containing PDA. Then the plates were cultured at 25°C, and the colony diameter was measured every 12 h to calculate the growth rate. After 10 days, the colony morphology of strain JZ1 was observed and photographed. To assess conidium production and morphology, the strains were cultured at 25°C for 10 days, and the conidia were collected and observed under a Nikon Eclipse Ci-S microscope. The pathogenicity test was conducted by spraying conidial suspension on maize leaves, which were then cultured at 25°C with 90% humidity for 7–10 days. Photographs were taken 10 days after inoculation. Each treatment consisted of more than three replicates, and experimental data were analyzed and visualized using R. Statistical significance was determined at *p* < 0.05 using Student’s *t*-test.

### 2.2 DNA extraction, PCR amplification and sequencing

The DNA of strains were extracted using the CTAB method (Allen et al., 2006). The DNA-ITS sequences of these strains were amplified using the primers ITS1 (5′-TCCGTAGGTGAACCTGCGG-3′) and ITS4 (5′-TCCTCCGCTTATTGATATGC-3′). The PCR products were submitted to a sequencing company for analysis, and sequence similarity was assessed by querying the GenBank database using BLASTn. Concurrently, we discerned unique genes from the genomes of *E. turcicum* an*d E. rostratum*, and employed the genes to design primers for validation. The gene sequences and primer sequences are listed in Supplementary Table S1.

### 2.3 Total RNA extraction, RNA-sequencing and mycoviruses confirmation

Strain JZ1 was cultured on PDA medium for 7 days, then the mycelium (1 g) was collected and ground to a powder in liquid nitrogen. Total RNA from strain JZ1 was extracted using RNAiso Plus following the instructions (Takara, Dalian, China). The extracted total RNA was utilized for metatranscriptomic sequencing, and virus-related contigs were obtained using previously described methods (Jia et al., 2021). To seek novel viruses or additional segments of known viruses, all assembled contigs exceeding 500 nt in length were analyzed. To confirm the presence of putative mycoviruses in the strain JZ1, the first-strand cDNA synthesis was performed using M-MLV reverse transcriptase with a hexdeoxyribonucleotide mixture of random primers (Takara, Dalian, China) according to the manufacturer’s instructions. Specific primers were designed according to the sequences of viral contigs, and PCR products were detected on 1% agarose gel electrophoresis.

### 2.4 dsRNA extraction and full-length cDNA cloning

Approximately 1 g of mycelium was collected from a cellophane membrane overlaying the PDA plate. The dsRNA from fungal mycelia was extracted using CF-11 cellulose (Sigma-Aldrich, Dorset, England) as previously described (Liu et al., 2014). The extracted dsRNA was utilized to obtain the terminal sequences of ErOrfV1 according to the single primer amplification method (Potgieter et al., 2009). The specific procedure was conducted as previously described (Mu et al., 2021).

### 2.5 Genome analysis, multiple alignment, and phylogenetic analysis

The full-length genomes of mycoviruses and putative ORFs were analyzed for their basic features using DNAMAN software (version 8.0; Lynnon Biosoft, Quebec, Canada). To elucidate the relationship between ErOrfV1 and previously reported orfanplasmoviruses, the RdRp and hypothetical protein (HP) sequences of known orfanplasmoviruses were obtained and subjected to multiple alignments using MAFFT ver. 7 (Katoh and Standley, 2013). The alignment results were then visualized using Jalview software. Subsequently, a percent identity matrix was generated for RdRp or HP coding sequences using R based on the results of the multiple sequence alignment. We used AlphaFold2 hosted through ColabFold to model the HP structure of orfanplasmoviruses (Mirdita et al., 2022). The generated PDB file was visualized using UCSF ChimeraX (version 1.7.1) (Pettersen et al., 2021).

Phylogenetic analysis was performed using representative RdRp sequences from members of the phyla *Lenarviricota*, *Kitrinoviricota*, *Pisuviricota* and unclassified narna-like viruses following methods described previously (Mu et al., 2018). For phylogenetic analysis, the aligned sequences were trimmed using trimAl with the -automated1 option (Capella-Gutierrez et al., 2009). The resulting trimmed multiple sequence alignment was then used to conduct a maximum-likelihood tree using IQ-TREE. The phylogenetic trees were visualized using FigTree (v 1.4.4) with midpoints rooted in increasing order. The multiple sequence alignment results (untrimmed and trimmed) and the phylogenetic tree files (in nwk format) can be found in Supplementary material S1.

### 2.6 Vertical and horizontal transmission of ErOrfV1

To assess the vertical transmission efficiency of ErOrfV1, conidial suspension from strain JZ1 was collected and spread on plates to obtain the single sub-isolates, yielding a total of 277 sub-isolates. Total RNA were extracted from these sub-isolates, followed by cDNA synthesis and mycovirus confirmation as described in 2.3.

For the evaluation of horizontal transmission, dual culture experiments were conducted. The virus-free strains of *E. rostratum*, *E. turcicum,* and *A. alternata* were chosen as the recipient strain, while strain JZ1 served as the donor strain. Co-cultures of the recipient and donor strains were grown on PDA plates (9 cm) for 10 days at 25°C. Subsequently, mycelial agar discs were obtained from the colony margin of the recipient strains and transferred to new PDA plates for further growth. After three plate-generations, RT-PCR was performed to detect the presence of the viruses.

## 3 Results

### 3.1 Isolation and identification of *Exserohilum rostratum* strain JZ1

**Figure 1:**
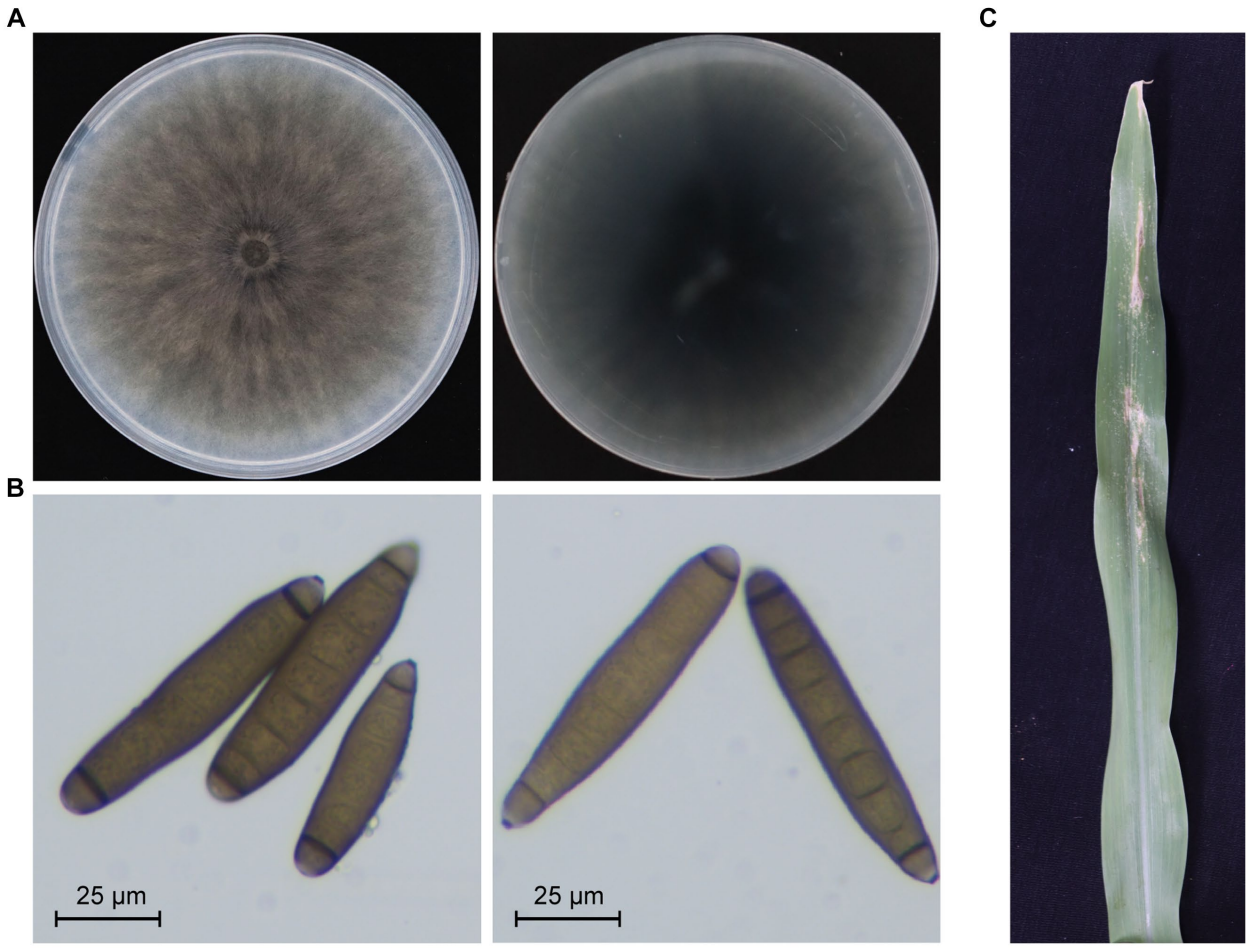
Biological characteristics of *Exserohilum rostratum* strain JZ1. **(A)** Colony morphology of strain JZ1. The left and right panels represent the front and back of the colony morphology, respectively. The strain was cultured on a PDA plate for 10 days at 25°C before photography. **(B)** Conidia morphology of *E. rostratum* JZ1. **(C)** Symptoms of maize leaf spot caused by strain JZ1. The image was captured 10 days post-inoculation with a conidial suspension at 25°C.

A strain designated as JZ1 was successfully isolated and purified from a diseased maize leaf in Shanxi province, China. The colony and conidiophore morphology of strain JZ1 were carefully observed on the PDA medium. After 10 days of cultivation, the colony of strain JZ1 exhibited a circular shape with a taupe color and light gray edging, while the reverse side of the colony appeared dark brown (Figure 1A). Examination of the conidiophores revealed fusiform structures with long ellipsoidal shapes, typically containing 6–10 septa. Each conidium displayed protruding basal hilum at both polar ends (Figure 1B). To confirm the identity of the pathogen isolated from maize, DNA-ITS sequences were performed on strain JZ1. The results of sequence analysis indicated a close genetic relationship between strain JZ1 and *E. rostratum* (Supplementary Figure S1). Pathogenicity tests further demonstrated that disease spots developed on maize leaves after 10 days of inoculation with strain JZ1, and subsequently, the strain JZ1 was successfully re-isolated from the lesions (Figure 1C). Based on morphological characteristics and sequence similarity, strain JZ1 was conclusively identified as *E. rostratum*.

### 3.2 Sequence analysis and genome organization of ErOrfV1

**Figure 2:**
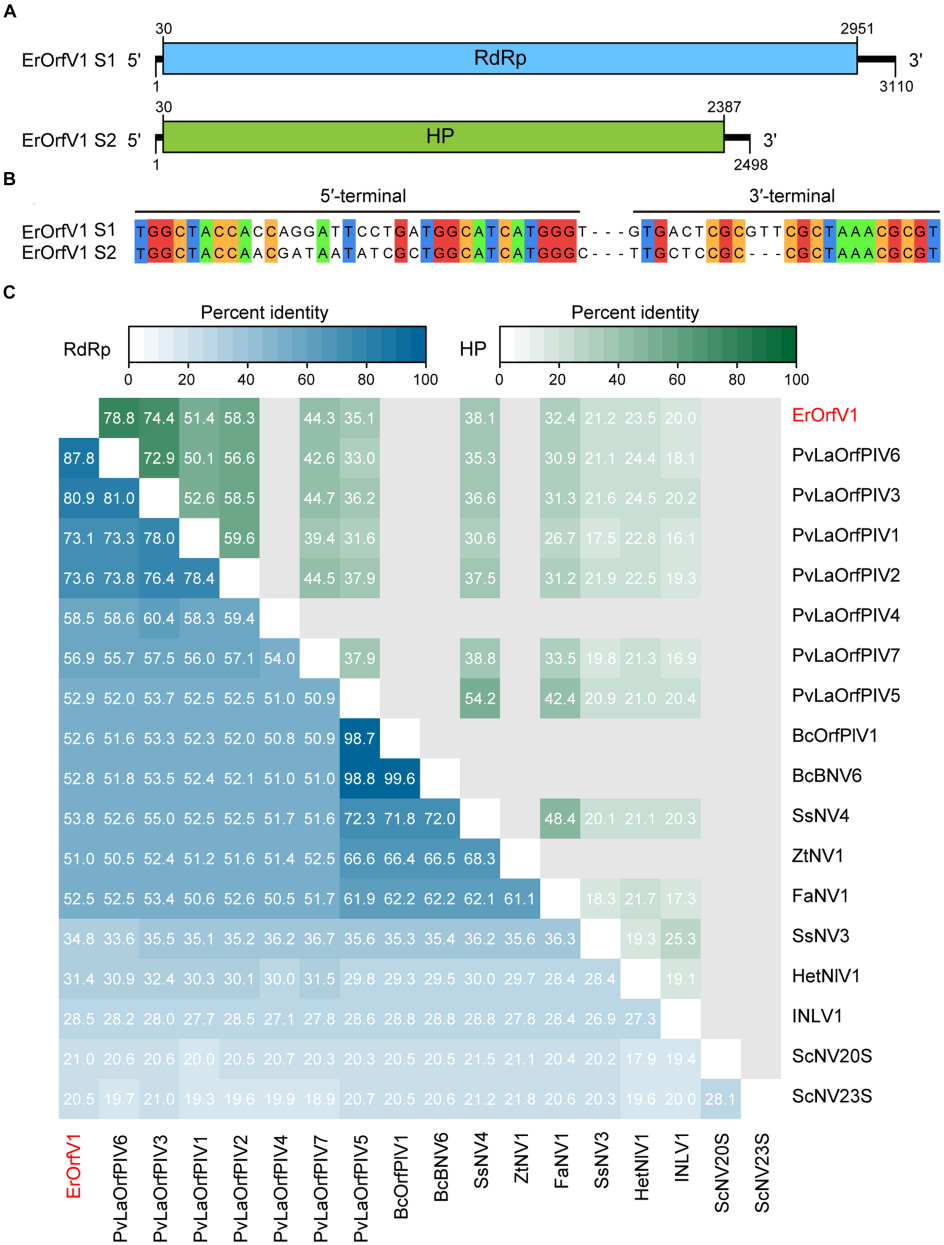
Genome organization of Exserohilum rostratum orfanplasmovirus 1 (ErOrfV1). **(A)** Schematic diagram of the ErOrfV1 genome. Open reading frames (ORFs) are depicted as boxes. **(B)** Multiple alignments of 5′- and 3′-terminal nucleotide sequences of the ErOrfV1 genomic segments. **(C)** Pairwise percent identity matrix of RNA-dependent RNA polymerases (RdRp) of ErOrfV1, *Saccharomyces cerevisiae* 20S, *Saccharomyces cerevisiae* 23S, and reported orfanplasmoviruses. The multiple alignments were performed using MAFFT ver.7, and the percent identity matrix was visualized using R. Abbreviations of viral names and GenBank accession numbers are listed in Supplementary Table S2.

To identify mycoviruses in strain JZ1, total RNA was extracted for RNA-sequencing. After analyzing all the assembled contigs that are over 500 nt in length, we discovered that the strain JZ1 was infected by a novel narna-like virus, which named Exserohilum rostratum orfanplasmovirus 1 (ErOrfV1) (GenBank Accession: PP350703 and PP350704). Aside from the sequences of ErOrfV1 and the *E. rostratum* genome, no additional contigs were identified. The complete genome of ErOrfV1 consists of two segments, and the sequences of 3′ and 5′ termini in both segments were highly conserved, confirming the acquisition of full-length sequences (Figures 2A,B). The sizes of the two segments were determined to be 3,110 nt and 2,498 nt, respectively, with each segment encoding a large ORF (ORF1 and ORF2) (Figure 2A). The protein encoded by ORF1 (30–2,951 nt) consisted of 973 amino acids with a size of 109.6 kDa. BLASTp analysis revealed the closest homology to the RdRp of Plasmopara viticola lesion associated orfanplasmovirus 6 (PvLaOrfPIV6; *E*-value = 0, identity = 87%). ORF2 (30–2,387 nt) was predicted to encode a HP of 785 amino acids with a size of 87.0 kDa, showing the highest similarity to the HP of PvLaOrfPIV6 (*E*-value = 0, identity = 78%).

To elucidate the relationship between ErOrfV1 and previously reported orfanplasmoviruses, a percent identity matrix was generated for RdRp or HP coding sequences using R. The analysis revealed that the RdRp of ErOrfV1 exhibited a sequence similarity ranging from 20.5 to 87.8% with reported orfanplasmoviruses, while the HP showed a similarity of 20.0 to 78.5%. Notably, ErOrfV1 shared significant sequence similarity with PvLaOrfPIV6 that was identified through RNA-sequencing (Figure 2C).

### 3.3 Phylogenetic analysis of ErOrfV1

**Figure 3:**
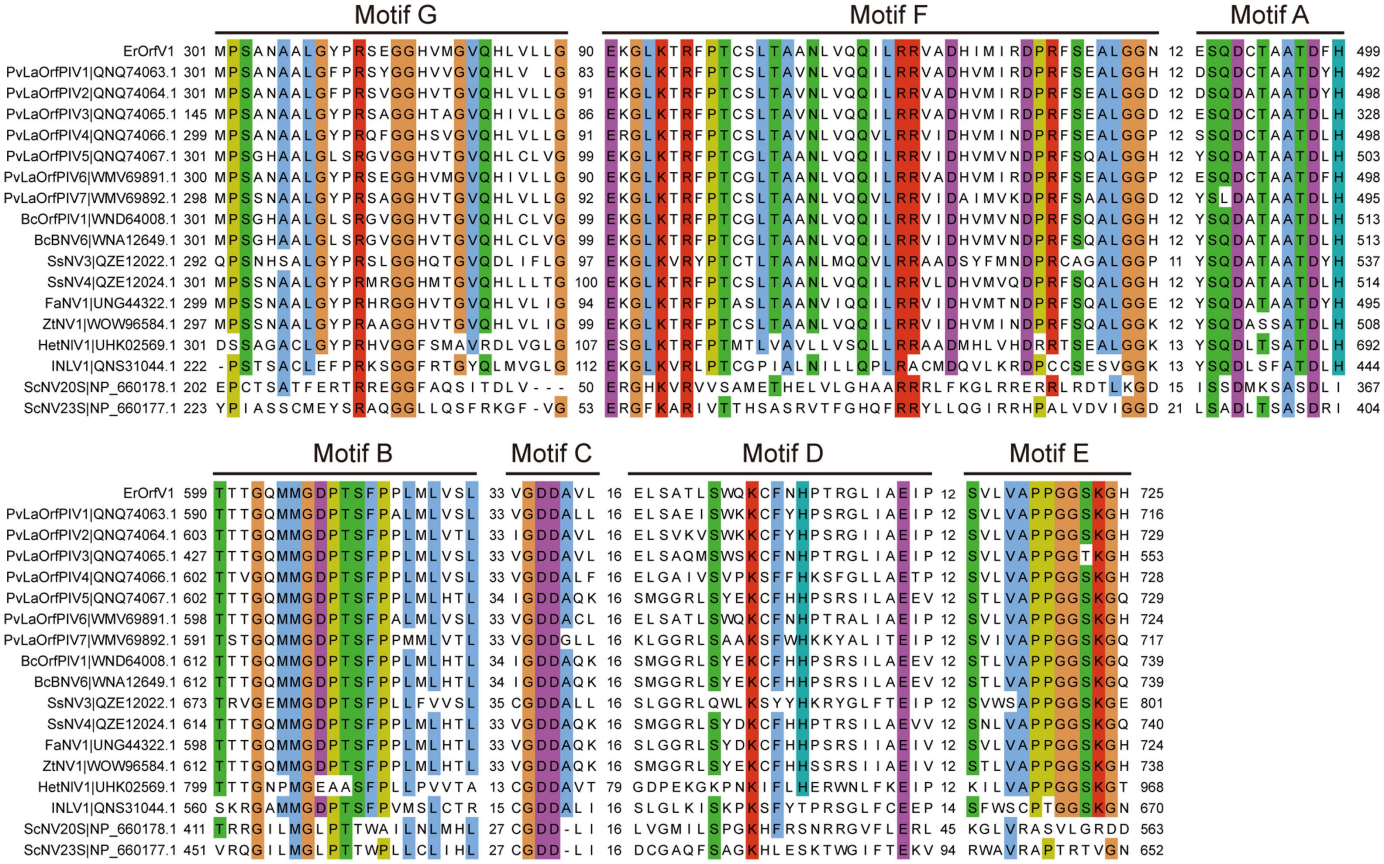
Alignment of ErOrfV1 and reported orfanplasmoviruses based on RdRp sequence. The alignment was generated using MAFFT. Conserved amino acids with 70% conservation are colored using the Cluster color mode in Jalview. Numbers refer to amino acids in the intervals between conserved seven motifs (G, F, A, B, C, D, E) were identified in the RdRp amino acid sequences. Abbreviations of viral names and GenBank accession numbers are listed in Supplementary Table S2.

**Figure 4:**
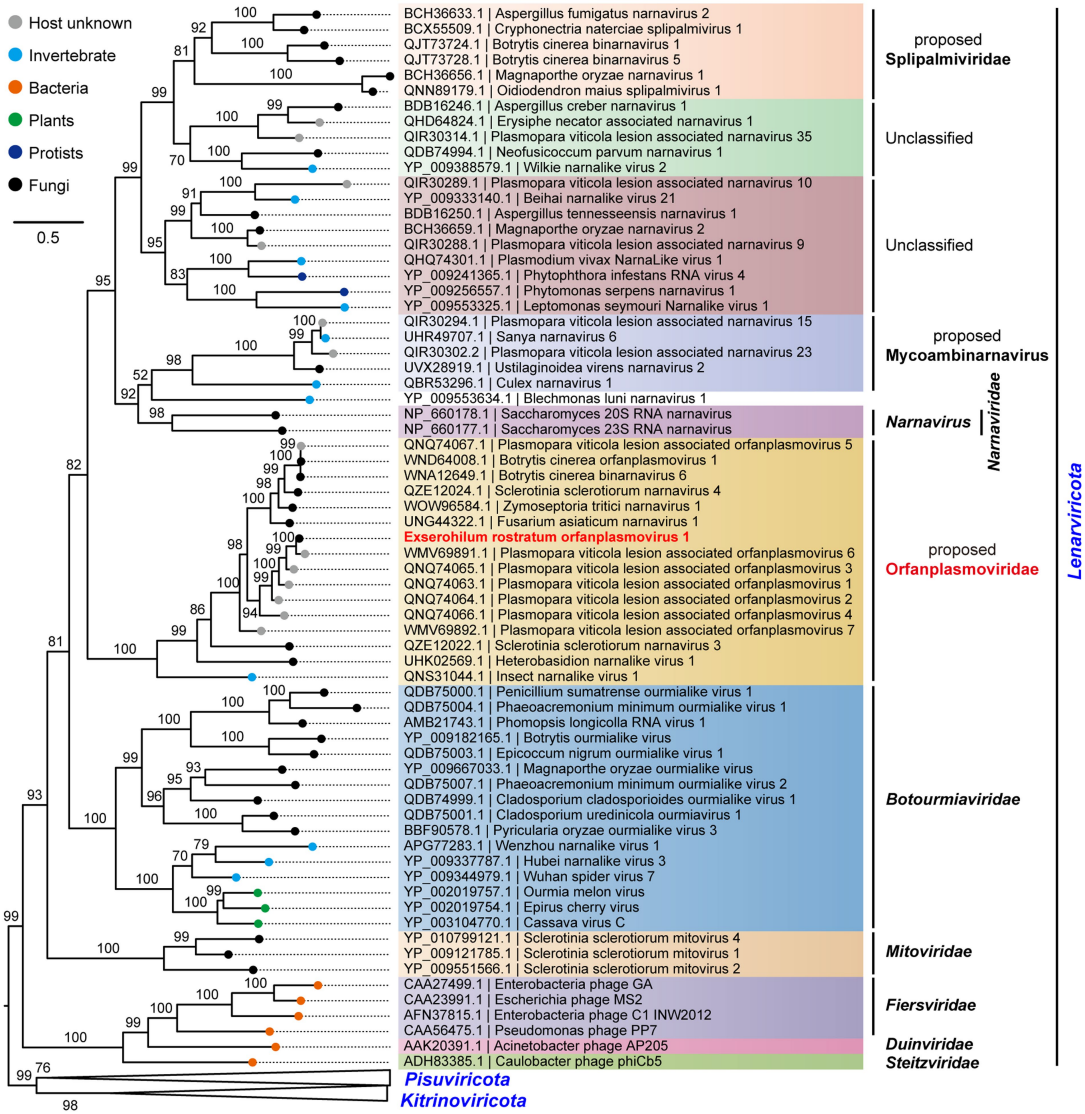
Phylogenetic tree of ErOrfV1 based on RdRp sequence. A maximum likelihood phylogenetic tree was constructed based on the alignment of the RdRp sequences. We performed a test for the best-fit amino acid substitution models using ModelFinder in IQ-TREE. According to the Bayesian Information Criterion, we found that the “PMB + R6” model is the most suitable for the data. Bootstrap values (%) obtained with 1,000 replicates are indicated on the branches, and branch lengths correspond to genetic distance. The phylogenetic trees were visualized using FigTree software. The virus ErOrfV1 is highlighted in red font.

To determine the evolutionary relationships of ErOrfV1 with other reported orfanplasmoviruses, multiple sequence alignments were performed using the RdRp amino acid sequence of ErOrfV1, along with sequences from known orfanplasmoviruses, and reference sequences ScNV20S and ScNV23S. The results revealed the presence of seven conserved motifs (motif G, F, A, B, C, D, and E) within the RdRp domain of ErOrfV1, including the characteristic “GDD” residues, which are typical signatures of (+)ssRNA viral RdRp in motif C (Figure 3). Based on the alignment results, a phylogenetic analysis was conducted, indicating that ErOrfV1 represents a novel orfanplasmovirus closely related to members of the family *Narnaviridae*. Interestingly, ErOrfV1 and known orfanplasmoviruses formed a distinct branch within the phylogenetic tree, suggesting the possibility of a novel taxon related to the family *Narnaviridae* (Figure 4 and Supplementary Figure S2).

Furthermore, multiple sequence alignments of the HP sequence revealed the presence of six conserved motifs (I-VI) shared among ErOrfV1 and other reported orfanplasmoviruses (Supplementary Figure S3). The similar structural features of these HP sequences suggest that these proteins may have analogous functions. We submitted the multiple sequence alignment file of the HP sequences to AlphaFold2 hosted by ColabFold for modeling. The results indicated that the HP proteins contains 23 α-helices and 15 β-sheets (Supplementary Figure S4). However, due to a lack of structural information for homologous proteins and an insufficient number of orfanplasmoviruses to compose a high-quality multiple sequence alignment file, the predicted 3D structure information may be inaccurate. We are not certain whether HP is the viral capsid protein, therefore we purified the virions according to the previous method (Wu et al., 2017). After lysing the extracted virion fractions and performing agarose gel electrophoresis, we did not observe distinct nucleic acid bands (Supplementary Figure S5A). The lysed nucleic acids were subjected to reverse transcription followed by PCR detection, through which the presence of the virus was confirmed (Supplementary Figure S5B). However, upon inspection of the purified virion fractions through transmission electron microscopy, neither viral particles nor structures resembling viral particles were observed. This arguably suggests that the virus might exist in a “naked” RNA form.

### 3.4 Vertical and horizontal transmission of ErOrfV1

**Figure 5:**
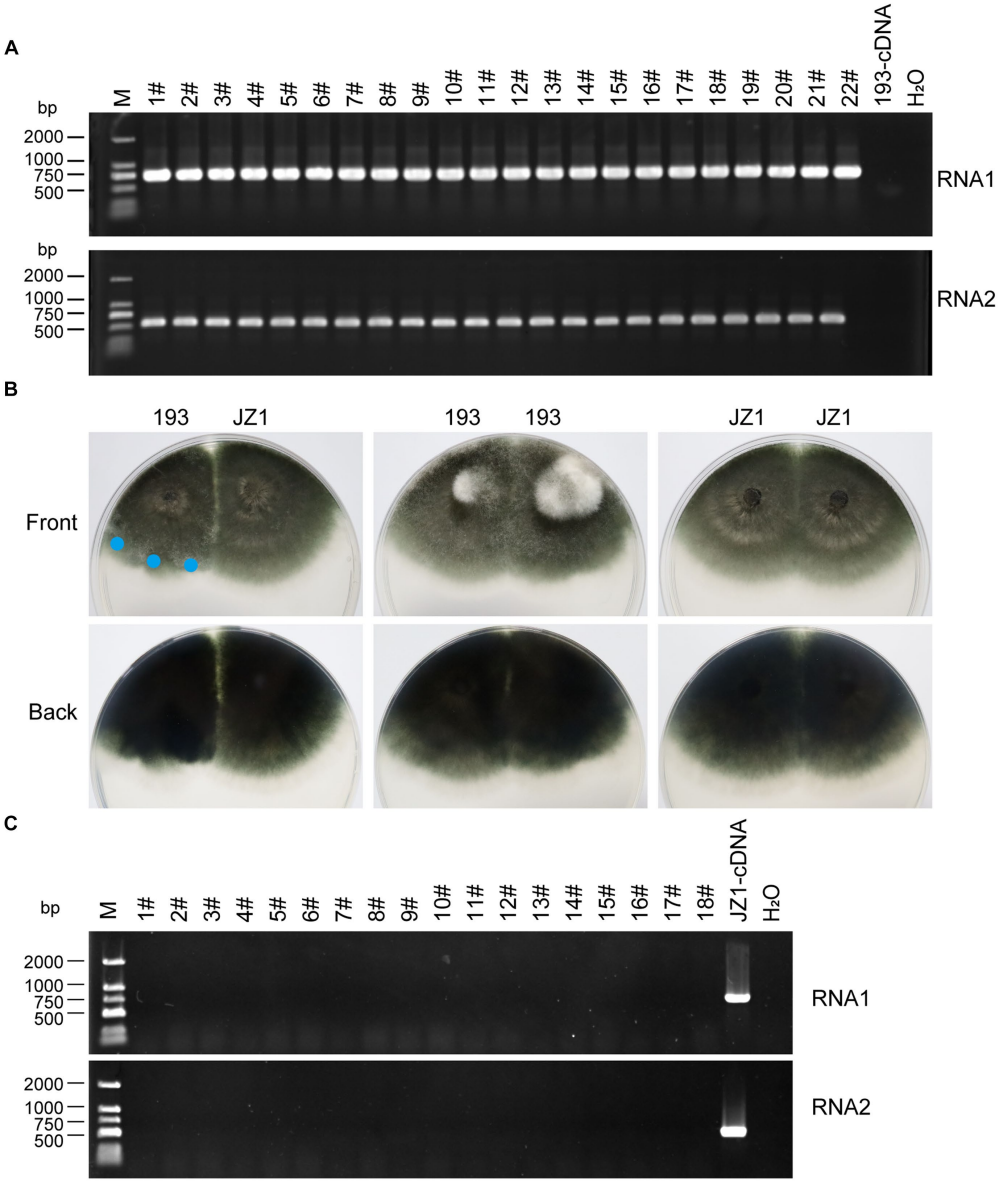
Vertical and horizontal transmission of ErOrfV1. **(A)** Vertical transmission of ErOrfV1. Single conidial sub-isolates were obtained from the original strain JZ1 and examined for the presence of the viruses by RT-PCR. Two pairs of primers targeting RNA1 and RNA2 segments of ErOrfV1 were used (Primers are listed in Supplementary Table S1). Lane M represents the DL-2000 Marker, numbers 1–22 with hashtags represent different conidial sub-isolated, 193-cDNA represents the cDNA of *E. rostratum* strain 193 without ErOrfV1. RNA1: the primers detected RNA1 segment of ErOrfV1 were used; RNA2: the primers detected RNA2 segment of ErOrfV1 were used. **(B)** Strain JZ1 was dual-cultured with a virus-free strain 193. Dual-cultures of strain 193 and 193, JZ1 and JZ1 served as the control. Photographs were taken after strains were co-cultured for 5 days at 25°C. Mycelial agar discs were taken from the colony margin of the recipient strain and transferred to another PDA plate (marked with a blue circle). Eighteen new isolates were obtained. **(C)** RT-PCR of the new isolates. After three plate-generations fungi, the RT-PCR was performed to detect the presence of viruses in the new isolates. Lane M represents the DL-2000 Marker, numbers 1–18 with hashtags representing new isolates from the recipient strains. JZ1-cDNA represents the cDNA of the strain JZ1.

The vertical transmission rate of ErOrfV1 was evaluated via single conidium isolation. A total of 277 sub-isolates were obtained, and the presence of ErOrfV1 was examined using specific primers (Supplementary Table S1). RT-PCR results revealed that all sub-isolates exhibited PCR products of the expected size for two segments of ErOrfV1, confirming that all sub-isolates were infected by ErOrfV1. In contrast, no corresponding bands were observed in the negative control *E. rostratum* strain 193 (Figure 5A). These results indicated that transmission of both RNA segments of ErOrfV1 to each sub-isolate indicated highly vertical transmissibility, with a transmission rate of 100% through conidia.

Subsequently, strain JZ1 was co-cultured with virus-free *E. rostratum* strain 193 to investigate the transmission rate of ErOrfV1 via hyphal fusion on PDA plates (Figure 5B). Mycelial agar plugs were picked up from colony margins, resulting in 18 strains after three rounds of sub-culturing. RT-PCR analysis of these strains revealed no detection of ErOrfV1 infection (Figure 5C). Moreover, compared to the dual-culture of control 193 with 193 or JZ1 with JZ1, a distinct barrier can be observed at the hyphal interaction region between the two strains after JZ1 has been dual-cultured with 193 for 5 days (Figure 5B). These findings suggest that ErOrfV1 cannot be transmitted to *E. rostratum* strain 193 via hyphal fusion, likely due to vegetative incompatibility.

### 3.5 Broad host range and possible asymptomatic infection by ErOrfV1

**Figure 6:**
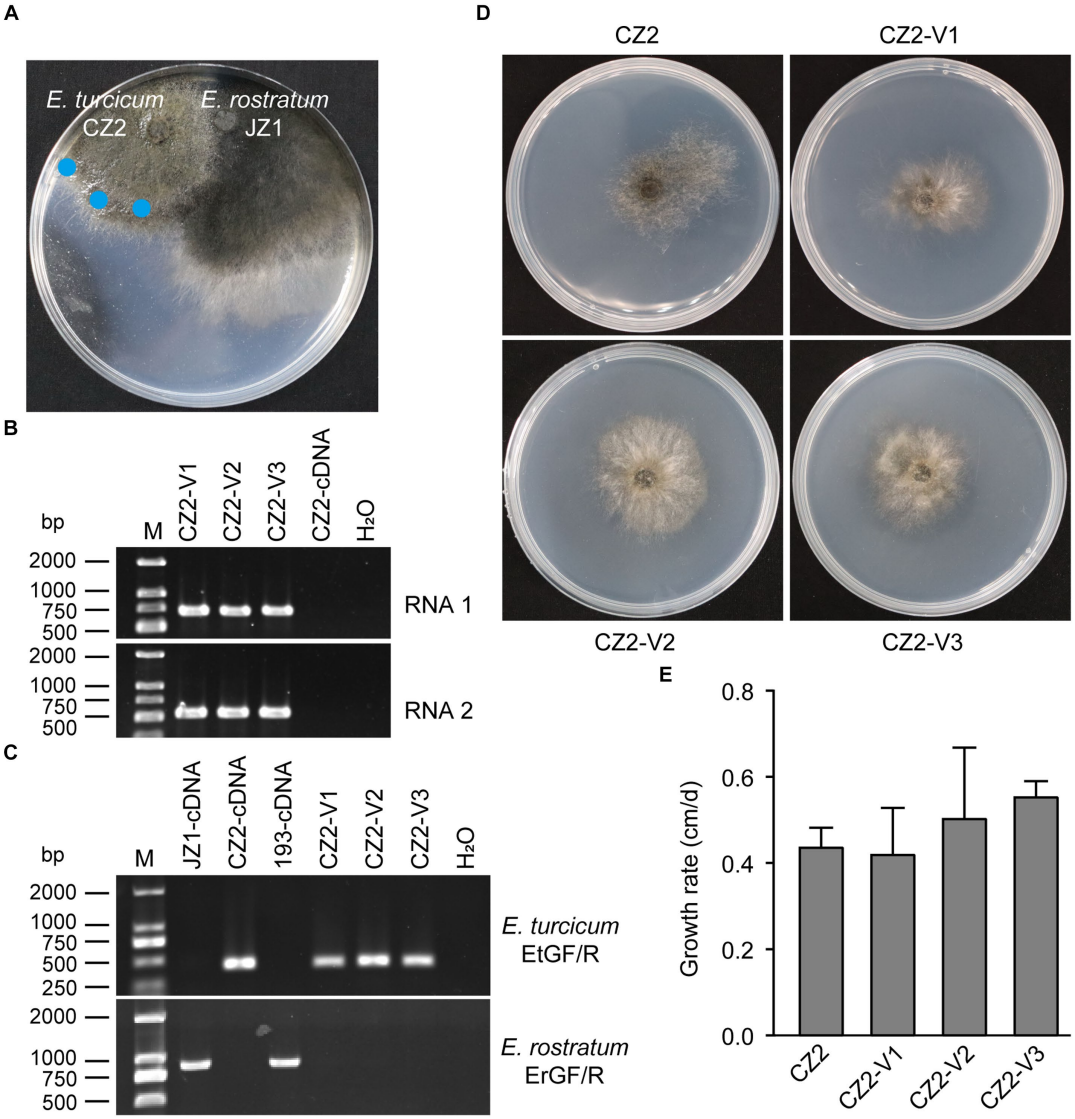
Horizontal transfer of ErOrfV1 in *E. turcicum*. **(A)** Strain JZ1 was dual cultured with *E. turcicum* strain CZ2. The strain JZ1 was co-cultured with *E. turcicum* strain for 10 days at 25°C. Mycelial agar discs were taken from the colony margin of the recipient strain and transferred to another PDA plate (marked with a blue circle). **(B)** RT-PCR detection of ErOrfV1. The primers used for detecting RNA1 and RNA2 are listed in Supplementary Table S1. CZ2-cDNA represents the cDNA of the *E. turcicum* strain CZ2. **(C)** Specific primers confirmation of *E. turcicum* and *E. rostratum* strains. The primers EtGF/R and ErGF/R were designed based on the specific genes from *E. turcicum* and *E. rostratum*, respectively. The primers and specific genes sequences are listed in Supplementary Table S1. **(D)** Colony morphology of *E. turcicum* strain CZ2 and *E. turcicum* new isolates CZ2-V1, V2, V3. These strains were cultured on a PDA plate for 7 days at 25°C before photography. **(E)** The growth rates of strains CZ2 and ErOrfV1-infected strains CZ2-V1, V2, V3. Based on the results of Student’s *t*-test, there was no significant difference in the growth rate at the *p* < 0.05 confidence level.

To assess the cross-species transmission of ErOrfV1 via hyphal fusion, strain JZ1 was cultured with *E. turcicum* and *A. alternata* on PDA plates (Figure 6A and Supplementary Figure S6). Following the intermingling of the two strains, mycelial agar discs were taken from the colonies of *E. turcicum* or *A. alternata* and transferred to another PDA plate for further growth. After three rounds of sub-culturing, the obtained strains were subjected to RT-PCR to detect ErOrfV1. Results indicated successful transmission of ErOrfV1 to *E. turcicum* via hyphal anastomoses, whereas transmission to *A. alternata* failed (Figure 6B and Supplementary Figure S6D). These virus-infected receptor isolates were confirmed to be pure *E. turcicum* strains using the RT-PCR method (Figure 6C) and ITS sequence (Supplementary material S2). Due to the unsuccessful attempts to obtain a virus-free strain of *E. rostratum* JZ1 via single conidial isolation, the effect of ErOrfV1 on *E. rostratum* could not be determined. However, strains of *E. turcicum* infected by ErOrfV1 were successfully obtained. Remarkably, the colony morphology and growth rate of *E. turcicum* infected by ErOrfV1 showed no significant difference compared to those of the original strains (Figures 6D,E), suggesting that ErOrfV1 may not significantly alter the phenotype of the host. It is noteworthy that we also identified a virus sequence with 98% nucleotide identity to ErOrfV1 in another strain 5-1-1 (GenBank Accession: PP554334 and PP554335) of *E. turcicum* isolated from maize leaves, indicating that cross-species transmission of ErOrfV1 has occurred in nature (Supplementary Figure S7).

## 4 Discussion

Metagenomic sequencing has revolutionized virus discovery, significantly expanding our understanding of the RNA virome (Neri et al., 2022). While many new viruses have been identified based on homology to known virus proteins in NCBI databases, a considerable portion of novel virus clades might remain elusive due to insufficient conservation of encoded proteins with known virus proteins. Chiapello et al. pioneered a distinct approach to identify ‘ORFan’ protein-encoding contigs, putatively viral, in lesions caused by the oomycete *Plasmopara viticola*, revealing five virus-like contigs. These contigs featured an ORF, and the encoded proteins exhibited some distant relationship to existing narnavirus RdRps, leading to the naming of these viruses as Plasmopara viticola orfanplasmoviruses 1–5, which formed a novel clade in the evolutionary tree (Chiapello et al., 2020). Subsequently, related novel viruses, including SsNV3, SsNV4, and FaNV1, were identified in *Sclerotinia sclerotiorum* and *Fusarium asiaticum*, respectively. Notably, the second segment of these viruses encodes a HP (Jia et al., 2021; Huang et al., 2023). Of the 15 reported orfanplasmoviruses, 11 possess a bi-segment genome, mirroring the genome organization of ErOrfV1, which also comprises two segments. Multiple alignments revealed conserved amino acid residues in these 11 proteins and the HP of ErOrfV1, suggesting a shared function (Supplementary Figure S3). Evolutionary analysis revealed a significant genetic distance between orfanplasmoviruses and the family *Narnaviridae*, resulting in the formation of a separate and distinct cluster. The genome of ErOrfV1 differs from known narnaviruses, and it exhibits considerable divergence from members of the family *Narnaviridae*. Therefore, we advocate for the establishment of a new family, Orfanplasmoviridae (intended as the members of the established phylum *Lenarviricota*, class *Amabiliviricetes*, order *Wolframvirales*), to encompass ErOrfV1 and its related, yet unidentified, orfanplasmoviruses.

Fifteen related virus sequences are currently available in the NCBI database, with only three viruses SsNV3, SsNV4, and FaNV1 having full-length sequences. This study successfully identified the full-length sequence of ErOrfV1, which holds significant importance for exploring the molecular characteristics of orfanplasmoviruses. Moreover, the biological aspects of orfanplasmoviruses, such as their infectivity, symptomatology, and transmissibility, remain poorly understood. This study aimed to address these gaps by investigating the horizontal and vertical transmission characteristics of ErOrfV1. The findings suggest that ErOrfV1 exhibits a high vertical transmission rate via conidia and is capable of cross-species transmission through hyphal anastomosis. Unfortunately, attempts to obtain a virus-free *E. rostratum* strain via multiple elimination and transfection methods were unsuccessful. However, the infection of ErOrfV1 in *E. turcicum* strains revealed an asymptomatic nature.

In our study, we observed that ErOrfV1 naturally infects *E. turcicum*. Given that both *E. rostratum* and *E. turcicum* can cause leaf spot disease in maize, and considering their similar ecological niches, it is conceivable that co-infection of maize by *E. rostratum* and *E. turcicum* could occur under field conditions (Xie et al., 2022; Xu et al., 2022). We hypothesize that cross-species transmission of ErOrfV1 may occur when maize plants are co-infected by both *E. rostratum* and *E. turcicum*. This phenomenon of cross-species transmission of mycoviruses is not uncommon in nature. For instance, Botrytis porri botybirnavirus 1 (BpBV1) was initially discovered in *Botrytis porri*, but subsequent studies found its sequence in other fungi such as *S. sclerotiorum* and *B. squamosa* (Wu et al., 2012; Mu et al., 2018; Wang et al., 2019). These three phytopathogenic fungi share similar ecological niches and thus have opportunities to co-infect the same plants. A similar scenario was observed in *R. necatrix* and *Fusarium* spp., where some viruses detected in *R. necatrix* were closely related to viruses infecting *Fusarium* spp. (Arjona-Lopez et al., 2018). Additionally, Deng et al. demonstrated cross-species transmission of Leptosphaeria biglobosa botybirnavirus 1 between *L. biglobosa* and *B. cinerea* (Deng et al., 2022). These findings suggest a potential mechanism for mycovirus transmission among different phytopathogenic fungi is potentially due to certain fungi with a wide host range acting as bridges or connectors (Deng et al., 2022). ErOrfV1 could potentially be transmitted from *E. rostratum* to *E. turcicum* through such a strategy, although the underlying mechanism requires further investigation.

In summary, our study identified ErOrfV1 as a novel bisegmented orfanplasmovirus, proposing a new family, Orfanplasmoviridae, related to the family *Narnaviridae*. We observed high vertical transmission rates of ErOrfV1 through conidia (277/277), while horizontal transmission through hyphal anastomosis in *E. rostratum* was not observed. Furthermore, our investigation of *E. turcicum* strains infected by ErOrfV1 revealed that ErOrfV1 does not affect the phenotype of *E. turcicum*. This study represents the first isolation of mycoviruses from the fungus *E. rostratum* and provides valuable insights into the biological and transmission characteristics of orfanplasmoviruses. Importantly, our findings significantly contribute to the diversity of viruses associated with *E. rostratum*.

## Data availability statement

The datasets presented in this study can be found in online repositories. The names of the repository/repositories and accession number(s) can be found at: https://www.ncbi.nlm.nih.gov/genbank/, PP350703; https://www.ncbi.nlm.nih.gov/genbank/, PP350704; https://www.ncbi.nlm.nih.gov/genbank/, PP554334; https://www.ncbi.nlm.nih.gov/genbank/, PP554335.

## Author contributions

JJ: Conceptualization, Funding acquisition, Visualization, Writing – original draft, Formal analysis. LN: Methodology, Visualization, Investigation, Writing – review & editing. ZS: Methodology, Visualization, Investigation, Writing – review & editing. XC: Investigation, Methodology, Writing – review & editing. JX: Investigation, Methodology, Writing – review & editing. LC: Investigation, Methodology, Writing – review & editing. BZ: Conceptualization, Resources, Writing – review & editing. FM: Conceptualization, Funding acquisition, Resources, Writing – review & editing, Writing – original draft.
