# Supplementary material for: Cross-species transmission of a novel bisegmented orfanplasmovirus in the phytopathogenic fungus Exserohilum rostratum
Source: Front Microbiol. 2024 May 23;15:1409677. doi: 10.3389/fmicb.2024.1409677 (PMC11153860; doi:10.3389/fmicb.2024.1409677)
Supplement: Supplementary file 4 [file Data_Sheet_2.ZIP › Multiple sequence alignment of ITS sequences.pdf]

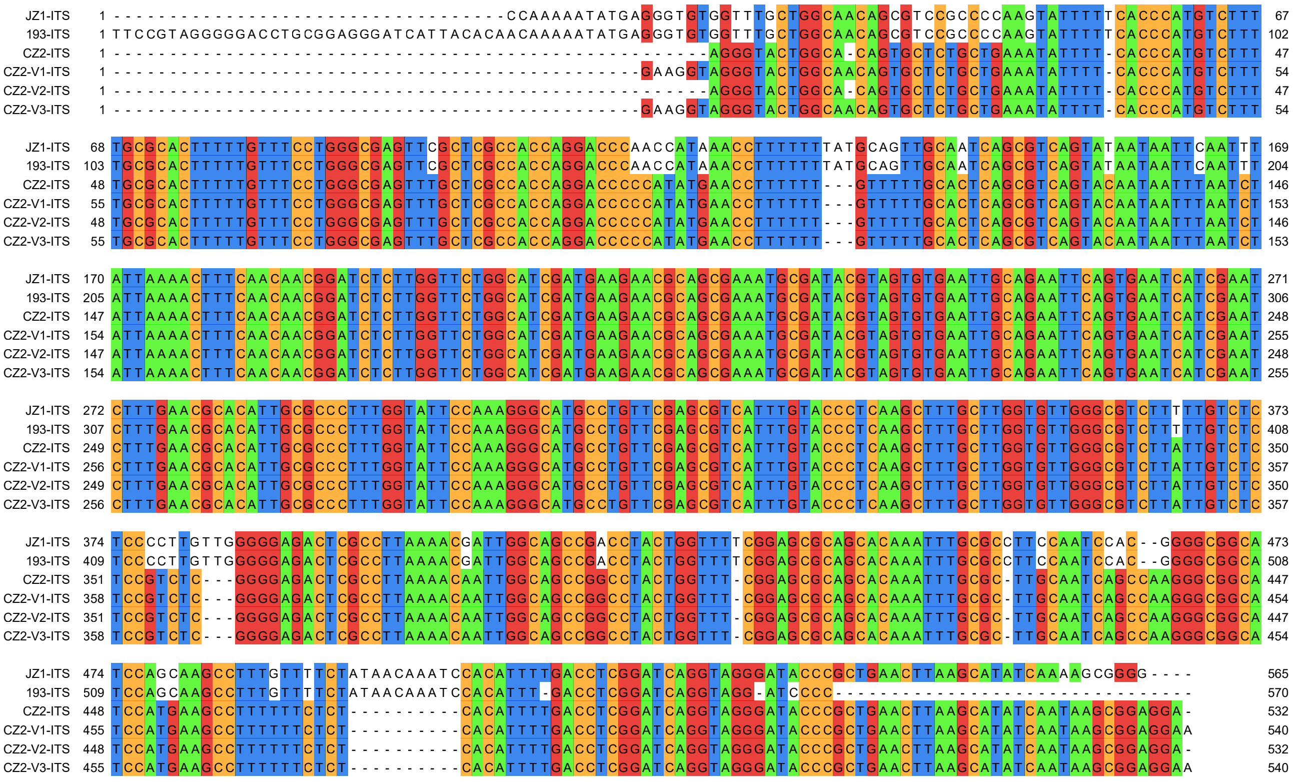

JZ1-ITS 1 -----------------------------------CCAAAAATATGAGGGTGTGGTTTGCTGGCAACAGCGTCCGCCCCAAGTATTTTTCACCCATGTCTTT 67
193-ITS 1 TTCCGTAGGGGGACCTGCGGAGGGATCATTACACAACAAAAATATGAGGGTGTGGTTTGCTGGCAACAGCGTCCGCCCCAAGTATTTTTCACCCATGTCTTT 102
CZ2-ITS 1 ---AGGGTACTGGCA-CAGTGCTCTGCTGAAATATTTT-CACCCATGTCTTT 47
CZ2-V1-ITS 1 ---GAAGGTAGGGTACTGGCAACAGTGCTCTGCTGAAATATTTT-CACCCATGTCTTT 54
CZ2-V2-ITS 1 ---AGGGTACTGGCA-CAGTGCTCTGCTGAAATATTTT-CACCCATGTCTTT 47
CZ2-V3-ITS 1 ---GAAGGTAGGGTACTGGCAACAGTGCTCTGCTGAAATATTTT-CACCCATGTCTTT 54
JZ1-ITS 68 TGCGCACTTTTTGTTTCCTGGGCGAGTTCGCTCGCCACCAGGACCCAACCATAAACCTTTTTTTATGCAGTTGCAATCAGCGTCAGTATAATAATTCAATTT 169
193-ITS 103 TGCGCACTTTTTGTTTCCTGGGCGAGTTCGCTCGCCACCAGGACCCAACCATAAACCTTTTTTTATGCAGTTGCAATCAGCGTCAGTATAATAATTCAATTT 204
CZ2-ITS 48 TGCGCACTTTTTGTTTCCTGGGCGAGTTTGCTCGCCACCAGGACCCCCATATGAACCTTTTTT---GTTTTTGCACTCAGCGTCAGTACAATAATTTAATCT 146
CZ2-V1-ITS 55 TGCGCACTTTTTGTTTCCTGGGCGAGTTTGCTCGCCACCAGGACCCCCATATGAACCTTTTTT---GTTTTTGCACTCAGCGTCAGTACAATAATTTAATCT 153
CZ2-V2-ITS 48 TGCGCACTTTTTGTTTCCTGGGCGAGTTTGCTCGCCACCAGGACCCCCATATGAACCTTTTTT---GTTTTTGCACTCAGCGTCAGTACAATAATTTAATCT 146
CZ2-V3-ITS 55 TGCGCACTTTTTGTTTCCTGGGCGAGTTTGCTCGCCACCAGGACCCCCATATGAACCTTTTTT---GTTTTTGCACTCAGCGTCAGTACAATAATTTAATCT 153
JZ1-ITS 170 ATTAAAACTTTCAACAACGGATCTCTTGGTTCTGGCATCGATGAAGAACGCAGCGAAATGCGATACGTAGTGTGAATTGCAGAATTCAGTGAATCATCGAAT 271
193-ITS 205 ATTAAAACTTTCAACAACGGATCTCTTGGTTCTGGCATCGATGAAGAACGCAGCGAAATGCGATACGTAGTGTGAATTGCAGAATTCAGTGAATCATCGAAT 306
CZ2-ITS 147 ATTAAAACTTTCAACAACGGATCTCTTGGTTCTGGCATCGATGAAGAACGCAGCGAAATGCGATACGTAGTGTGAATTGCAGAATTCAGTGAATCATCGAAT 248
CZ2-V1-ITS 154 ATTAAAACTTTCAACAACGGATCTCTTGGTTCTGGCATCGATGAAGAACGCAGCGAAATGCGATACGTAGTGTGAATTGCAGAATTCAGTGAATCATCGAAT 255
CZ2-V2-ITS 147 ATTAAAACTTTCAACAACGGATCTCTTGGTTCTGGCATCGATGAAGAACGCAGCGAAATGCGATACGTAGTGTGAATTGCAGAATTCAGTGAATCATCGAAT 248
CZ2-V3-ITS 154 ATTAAAACTTTCAACAACGGATCTCTTGGTTCTGGCATCGATGAAGAACGCAGCGAAATGCGATACGTAGTGTGAATTGCAGAATTCAGTGAATCATCGAAT 255
JZ1-ITS 272 CTTTGAACGCACATTGCGCCCTTTGGTATTCCAAAGGGCATGCCTGTTCGAGCGTCATTTGTACCCTCAAGCTTTGCTTGGTGTTGGGCGTCTTTTTGTCTC 373
193-ITS 307 CTTTGAACGCACATTGCGCCCTTTGGTATTCCAAAGGGCATGCCTGTTCGAGCGTCATTTGTACCCTCAAGCTTTGCTTGGTGTTGGGCGTCTTTTTGTCTC 408
CZ2-ITS 249 CTTTGAACGCACATTGCGCCCTTTGGTATTCCAAAGGGCATGCCTGTTCGAGCGTCATTTGTACCCTCAAGCTTTGCTTGGTGTTGGGCGTCTTATTGTCTC 350
CZ2-V1-ITS 256 CTTTGAACGCACATTGCGCCCTTTGGTATTCCAAAGGGCATGCCTGTTCGAGCGTCATTTGTACCCTCAAGCTTTGCTTGGTGTTGGGCGTCTTATTGTCTC 357
CZ2-V2-ITS 249 CTTTGAACGCACATTGCGCCCTTTGGTATTCCAAAGGGCATGCCTGTTCGAGCGTCATTTGTACCCTCAAGCTTTGCTTGGTGTTGGGCGTCTTATTGTCTC 350
CZ2-V3-ITS 256 CTTTGAACGCACATTGCGCCCTTTGGTATTCCAAAGGGCATGCCTGTTCGAGCGTCATTTGTACCCTCAAGCTTTGCTTGGTGTTGGGCGTCTTATTGTCTC 357
JZ1-ITS 374 TCCCCTTGTTGGGGGAGACTCGCCTTAAAACGATTGGCAGCCGACCTACTGGTTTTCGGAGCGCAGCACAAATTTGCGCCTTCCAATCCAC--GGGGCGGCA 473
193-ITS 409 TCCCCTTGTTGGGGGAGACTCGCCTTAAAACGATTGGCAGCCGACCTACTGGTTTTCGGAGCGCAGCACAAATTTGCGCCTTCCAATCCAC--GGGGCGGCA 508
CZ2-ITS 351 TCCGTCTC---GGGGAGACTCGCCTTAAAACAATTGGCAGCCGGCCTACTGGTTT-CGGAGCGCAGCACAAATTTGCGC-TTGCAATCAGCCAAGGGCGGCA 447
CZ2-V1-ITS 358 TCCGTCTC---GGGGAGACTCGCCTTAAAACAATTGGCAGCCGGCCTACTGGTTT-CGGAGCGCAGCACAAATTTGCGC-TTGCAATCAGCCAAGGGCGGCA 454
CZ2-V2-ITS 351 TCCGTCTC---GGGGAGACTCGCCTTAAAACAATTGGCAGCCGGCCTACTGGTTT-CGGAGCGCAGCACAAATTTGCGC-TTGCAATCAGCCAAGGGCGGCA 447
CZ2-V3-ITS 358 TCCGTCTC---GGGGAGACTCGCCTTAAAACAATTGGCAGCCGGCCTACTGGTTT-CGGAGCGCAGCACAAATTTGCGC-TTGCAATCAGCCAAGGGCGGCA 454
JZ1-ITS 474 TCCAGCAAGCCTTTGTTTTCTATAACAAATCCACATTTTGACCTCGGATCAGGTAGGGATACCCGCTGAACTTAAGCATATCAAAAGCGGG---- 565
193-ITS 509 TCCAGCAAGCCTTTGTTTTCTATAACAAATCCACATTT-GACCTCGGATCAGGTAGG-ATCCCC------------------------------- 570
CZ2-ITS 448 TCCATGAAGCCTTTTTTCTCT-----------CACATTTTGACCTCGGATCAGGTAGGGATACCCGCTGAACTTAAGCATATCAATAAGCGGAGGA- 532
CZ2-V1-ITS 455 TCCATGAAGCCTTTTTTCTCT-----------CACATTTTGACCTCGGATCAGGTAGGGATACCCGCTGAACTTAAGCATATCAATAAGCGGAGGAA 540
CZ2-V2-ITS 448 TCCATGAAGCCTTTTTTCTCT-----------CACATTTTGACCTCGGATCAGGTAGGGATACCCGCTGAACTTAAGCATATCAATAAGCGGAGGA- 532
CZ2-V3-ITS 455 TCCATGAAGCCTTTTTTCTCT-----------CACATTTTGACCTCGGATCAGGTAGGGATACCCGCTGAACTTAAGCATATCAATAAGCGGAGGAA 540
