# Supplementary material for: Cross-species transmission of a novel bisegmented orfanplasmovirus in the phytopathogenic fungus Exserohilum rostratum
Source: Front Microbiol. 2024 May 23;15:1409677. doi: 10.3389/fmicb.2024.1409677 (PMC11153860; doi:10.3389/fmicb.2024.1409677)
Supplement: Supplementary file 5 [file Data_Sheet_3.DOCX]

Supplementary Material

# Supplementary figure legends

**Supplementary Figure 1. The blast result of ITS sequence of strain JZ1.**

**Supplementary Figure 2. Phylogenetic tree of ErOrfV1 based on RdRp sequence.** A maximum likelihood phylogenetic tree was constructed based on the alignment of the RdRp sequence.

**Supplementary Figure 3. Alignment of ErOrfV1 and reported orfanplasmoviruses based on HP sequence.** The alignment was generated using MAFFT. The conserved amino acids with 70% conservation are colored in Cluster color mode in Jalview.

**Supplementary Figure 4. The structural informations of the HP in orfanplasmoviruses.** (A) AlphaFold2 predicted 3D structure of HP. (B) Secondary structure representation of the multiple sequence alignment of the HP, rendered using ESPript 3.0.

**Supplementary Figure 5. The virus particle extraction.** (A) Electrophoretic profile of viral RNA extracted from different fractions after sugar density gradient centrifugation. (B) RT-PCR of the viral RNA extracted from different fractions after sugar density gradient centrifugation. The viral RNA extraction was performed using M-MLV reverse transcriptase with a hexdeoxyribonucleotide mixture of random primers (Takara, Dalian, China) according to the manufacturer's instructions. The primers used for detecting RNA1 and RNA2 are listed in Supplementary Table S1.

**Supplementary Figure 6.** **Horizontal transfer of ErOrfV1 in *E. turcicum* or *Alternaria alternata*.** (A) Strain JZ1 was dual cultured with *E. turcicum* strain CZ2. (B) Strain CZ2 dual-cultured with CZ2 was as control. Photographs were taken after strains were co-cultured for 5 days at 25°C. (C) Strain JZ1 was dual cultured with *A. alternata* strain. The strain JZ1 was co-cultured with *A. alternata* strain for 10 days at 25°C. Mycelial agar discs were taken from the colony margin of the recipient strain and transferred to another PDA plate (marked with a blue circle), and 15 new isolates were obtained. (D) RT-PCR amplification of new *A. alternata* isolates. After three plate-generations fungi, the RT-PCR was performed to detect the presence of viruses in the new isolates. Lane M represents the DL-2000 Marker, numbers 1-15 with hashtags representing new isolates from the recipient strains. JZ1-cDNA represents the cDNA of the strain JZ1.

**Supplementary Figure 7. RT-PCR amplification of ErOrfV1 in the strain 5-1-1.** (A). Colony morphology of *E. turcicum* strain 5-1-1. The strain was cultured on a PDA plate for 10 days at 25°C before photography. (B). RT-PCR amplification of strain 5-1-1. The RT-PCR was performed to detect the presence of viruses in the strain 5-1-1. Lane M represents the DL-2000 Marker, 5-1-1 represents the cDNA of the strain JZ1, and JZ1 represents the cDNA of the strain JZ1.

**Supplementary Table1. The primers used for the detection of ErOrfV1.**

**Supplementary Table 2. The information of viruses used in multiple alignments.**

**Supplementary Material 1. The multiple alignment and phylogenetic tree files for Figure 4.**

**Supplementary Material 2.** **The ITS sequence of strain JZ1, 193, CZ2 and CZ2-V1, V2, V3.**
