# Supplementary material for: Cross-species transmission of a novel bisegmented orfanplasmovirus in the phytopathogenic fungus Exserohilum rostratum
Source: Front Microbiol. 2024 May 23;15:1409677. doi: 10.3389/fmicb.2024.1409677 (PMC11153860; doi:10.3389/fmicb.2024.1409677)
Supplement: Supplementary file 6 [file Image_1.PDF]

BLAST ® » blastn suite » RID-A9H80SBA016

BLAST Results

Questions/comments

Job title: YCBT

**RID**	A9H80SBA016 (Expires on 07-06 09:18 am)
**Query ID**	lcl|Query_25567
**Description**	YCBT
**Molecule type**	dna
**Query Length**	563
**Database Name**	nt
**Description**	Nucleotide collection (nt)
**Program**	BLASTN 2.14.1+

Graphic Summary

Distribution of the top 100 Blast Hits on 100 subject sequences

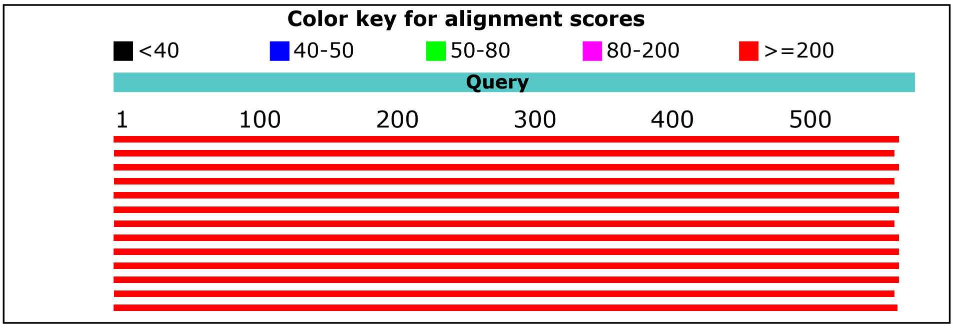

Descriptions

Description	Scientific Name	Max Score	Total Score	Query Cover	E value	Per. Ident	Acc. Len	Accession
Exserohilum rostratum isolate SDFG3C7 internal transcribed spacer 1, partial sequence; 5.8S ribosomal RNA gene and internal transcribed spacer 2, complete sequence; and large subunit ribosomal RNA gene, partial sequence	Exserohilum rostratum	1040	1040	100%	0.0	100.00%	607	MT322140.1
Exserohilum rostratum isolate SDFG3C10 internal transcribed spacer 1, partial sequence; 5.8S ribosomal RNA gene and internal transcribed spacer 2, complete sequence; and large subunit ribosomal RNA gene, partial sequence	Exserohilum rostratum	1035	1035	99%	0.0	100.00%	609	MT322143.1
Exserohilum rostratum isolate SDFG3C2 internal transcribed spacer 1, partial sequence; 5.8S ribosomal RNA gene and internal transcribed spacer 2, complete sequence; and large subunit ribosomal RNA gene, partial sequence	Exserohilum rostratum	1035	1035	100%	0.0	99.82%	593	MT322135.1
Exserohilum rostratum isolate SDFG3B7 internal transcribed spacer 1, partial sequence; 5.8S ribosomal RNA gene and internal transcribed spacer 2, complete sequence; and large subunit ribosomal RNA gene, partial sequence	Exserohilum rostratum	1035	1035	99%	0.0	100.00%	589	MT322129.1
Exserohilum rostratum isolate W204 internal transcribed spacer 1, partial sequence; 5.8S ribosomal RNA gene and internal transcribed spacer 2, complete sequence; and large subunit ribosomal RNA gene, partial sequence	Exserohilum rostratum	1035	1035	100%	0.0	99.82%	625	MN599628.1

Alignments

Alignment view Pairwise ☐ CDS feature Restore defaults

Exserohilum rostratum isolate SDFG3C7 internal transcribed spacer 1, partial sequence; 5.8S ribosomal RNA gene and internal transcribed spacer 2, complete sequence; and large subunit ribosomal RNA gene, partial sequence

Sequence ID: **MT322140.1** Length: 607 Number of Matches: 1
Range 1: 40 to 602

Score	Expect	Identities	Gaps	Strand	Frame
1040 bits(563)	0.0()	563/563(100%)	0/563(0%)	Plus/Plus	

```
Query  1    CAAAAATATGAGGGTGTGGTTTGCTGGCAACAGCGTCCGCCCCAAGTATTTTTCACCCAT  60
            ||||||||||||||||||||||||||||||||||||||||||||||||||||||||||||
Sbjct  40   CAAAAATATGAGGGTGTGGTTTGCTGGCAACAGCGTCCGCCCCAAGTATTTTTCACCCAT  99

Query  61   GTCTTTTGCGCACTTTTTGTTTCCTGGGCGAGTTCGCTCGCCACCAGGACCCAACCATAA  120
            ||||||||||||||||||||||||||||||||||||||||||||||||||||||||||||
Sbjct  100  GTCTTTTGCGCACTTTTTGTTTCCTGGGCGAGTTCGCTCGCCACCAGGACCCAACCATAA  159

Query  121  ACCtttttttATGCAGTTGCAATCAGCGTCAGTATAATAATTCAATTTATTAAAACTTTC  180
            ||||||||||||||||||||||||||||||||||||||||||||||||||||||||||||
Sbjct  160  ACCTTTTTTTATGCAGTTGCAATCAGCGTCAGTATAATAATTCAATTTATTAAAACTTTC  219

Query  181  AACAACGGATCTCTTGGTTCTGGCATCGATGAAGAACGCAGCGAAATGCGATACGTAGTG  240
            ||||||||||||||||||||||||||||||||||||||||||||||||||||||||||||
Sbjct  220  AACAACGGATCTCTTGGTTCTGGCATCGATGAAGAACGCAGCGAAATGCGATACGTAGTG  279

Query  241  TGAATTGCAGAATTCAGTGAATCATCGAATCTTTGAACGCACATTGCGCCCTTTGGTATT  300
            ||||||||||||||||||||||||||||||||||||||||||||||||||||||||||||
Sbjct  280  TGAATTGCAGAATTCAGTGAATCATCGAATCTTTGAACGCACATTGCGCCCTTTGGTATT  339

Query  301  CCAAAGGGCATGCCTGTTCGAGCGTCATTTGTACCCTCAAGCTTTGCTTGGTGTTGGGCG  360
            ||||||||||||||||||||||||||||||||||||||||||||||||||||||||||||
Sbjct  340  CCAAAGGGCATGCCTGTTCGAGCGTCATTTGTACCCTCAAGCTTTGCTTGGTGTTGGGCG  399

Query  361  TCTTTTTGTCTCTCCCCTTGTTGGGGGAGACTCGCCTTAAAACGATTGGCAGCCGACCTA  420
            ||||||||||||||||||||||||||||||||||||||||||||||||||||||||||||
Sbjct  400  TCTTTTTGTCTCTCCCCTTGTTGGGGGAGACTCGCCTTAAAACGATTGGCAGCCGACCTA  459

Query  421  CTGGTTTTCGGAGCGCAGCACAAATTTGCGCCTTCCAATCCACGGGGCGGCATCCAGCAA  480
            ||||||||||||||||||||||||||||||||||||||||||||||||||||||||||||
Sbjct  460  CTGGTTTTCGGAGCGCAGCACAAATTTGCGCCTTCCAATCCACGGGGCGGCATCCAGCAA  519

Query  481  GCCTTTGTTTTCTATAACAAATCCACATTTTGACCTCGGATCAGGTAGGGATACCCGCTG  540
            ||||||||||||||||||||||||||||||||||||||||||||||||||||||||||||
Sbjct  520  GCCTTTGTTTTCTATAACAAATCCACATTTTGACCTCGGATCAGGTAGGGATACCCGCTG  579

Query  541  AACTTAAGCATATCAAAAGCGGG  563
            |||||||||||||||||||||||
Sbjct  580  AACTTAAGCATATCAAAAGCGGG  602
```
