# Supplementary figures and images for: Cross-species transmission of a novel bisegmented orfanplasmovirus in the phytopathogenic fungus Exserohilum rostratum
Source: Front Microbiol. 2024 May 23;15:1409677. doi: 10.3389/fmicb.2024.1409677 (PMC11153860; doi:10.3389/fmicb.2024.1409677)

- Host unknown  
● Invertebrate  
● Bacteria  
● Plants  
● Protists  
● Fungi

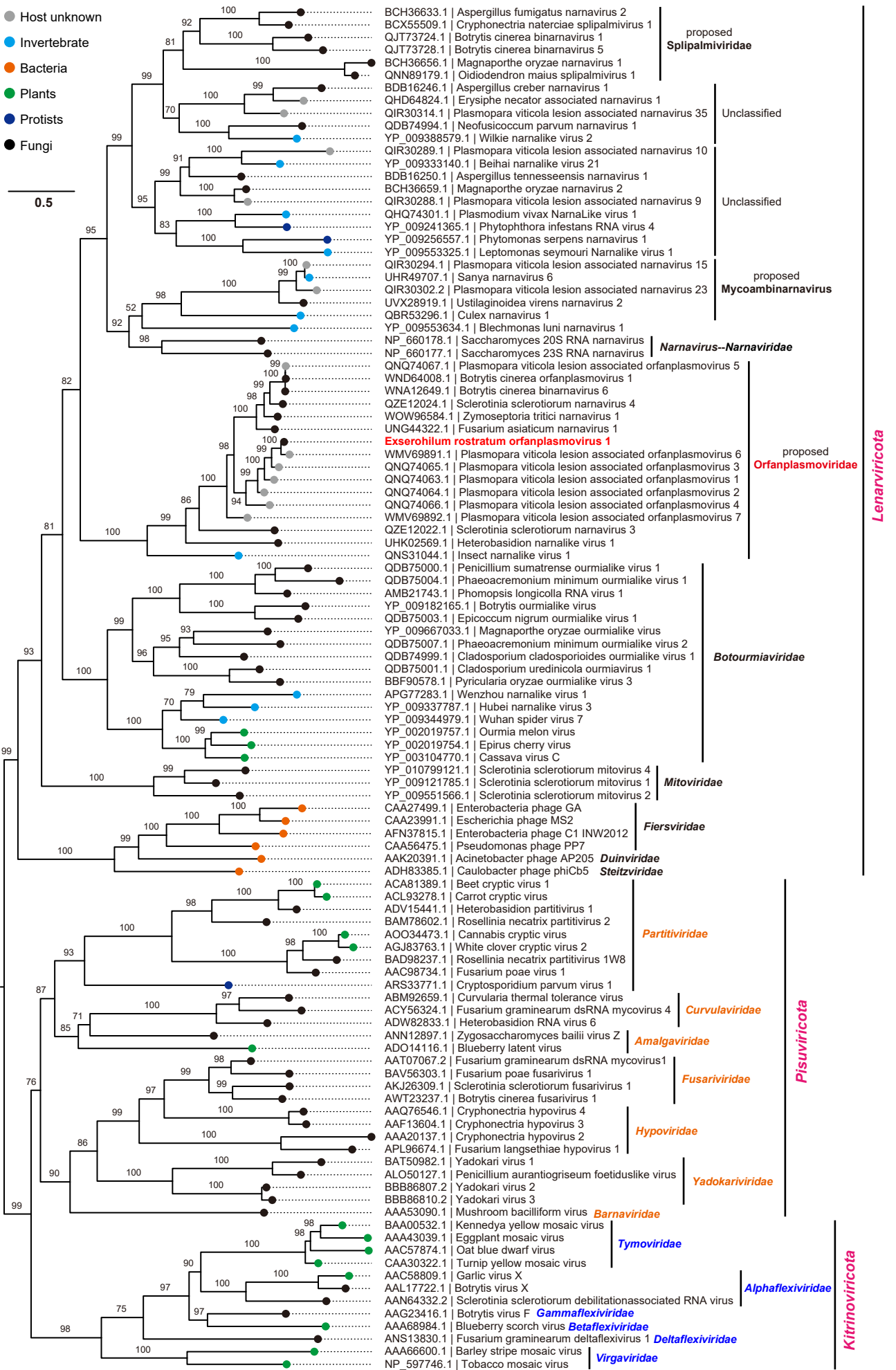

Supplement: Supplementary file 7 [file Image_2.PDF]

**A**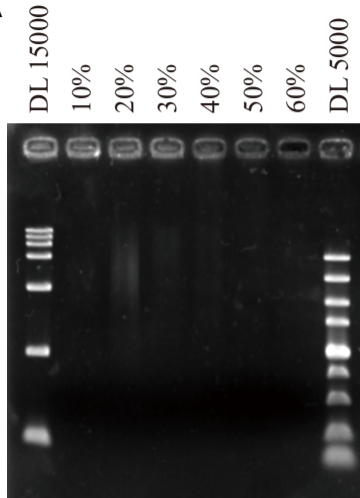**B**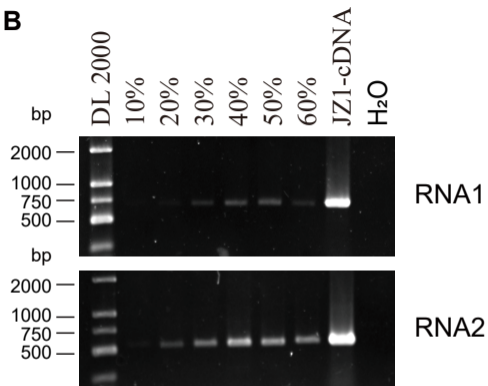

Supplement: Supplementary file 10 [file Image_5.PDF]

**A**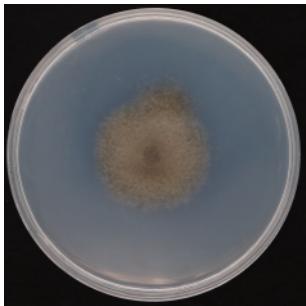

*E. turcicum* 5-1-1

**B**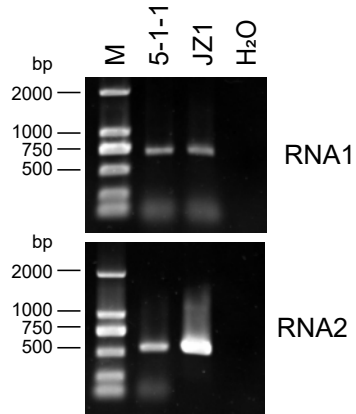

Supplement: Supplementary file 12 [file Image_7.PDF]
