# Supplementary material for: Cross-species transmission of a novel bisegmented orfanplasmovirus in the phytopathogenic fungus Exserohilum rostratum
Source: Front Microbiol. 2024 May 23;15:1409677. doi: 10.3389/fmicb.2024.1409677 (PMC11153860; doi:10.3389/fmicb.2024.1409677)
Supplement: Supplementary file 8 [file Image_3.PDF]

A

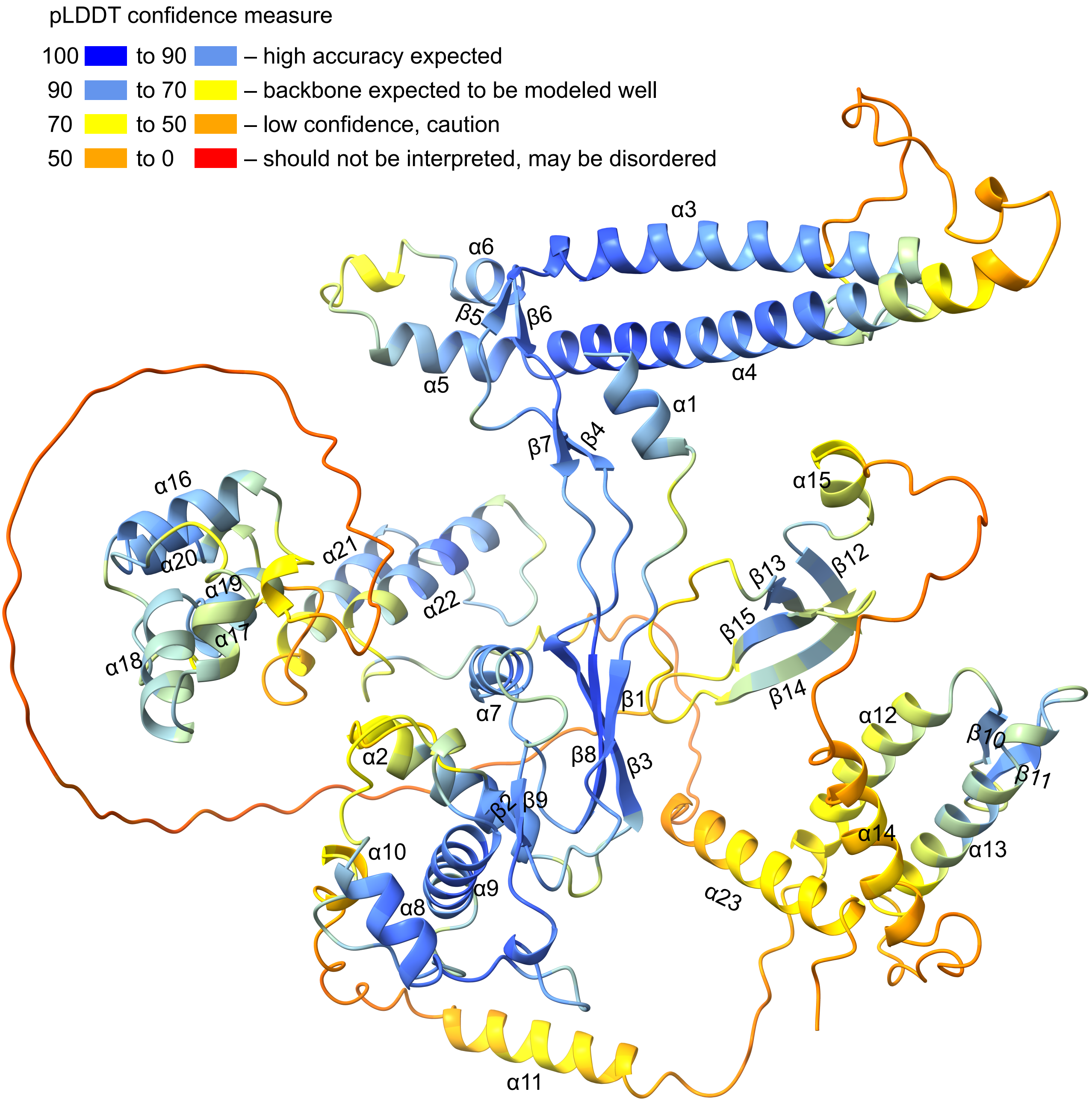
pLDDT confidence measure
100 to 90 – high accuracy expected
90 to 70 – backbone expected to be modeled well
70 to 50 – low confidence, caution
50 to 0 – should not be interpreted, may be disordered
α3
α6
β5
β6
α5
α4
α1
β7
β4
α15
α16
α20
α21
α19
α22
β13
β12
β15
α17
α18
α7
β1
β14
α12
α2
β8
β3
β10
β11
β2
β9
α10
α14
α13
α9
α8
α23
α11

B

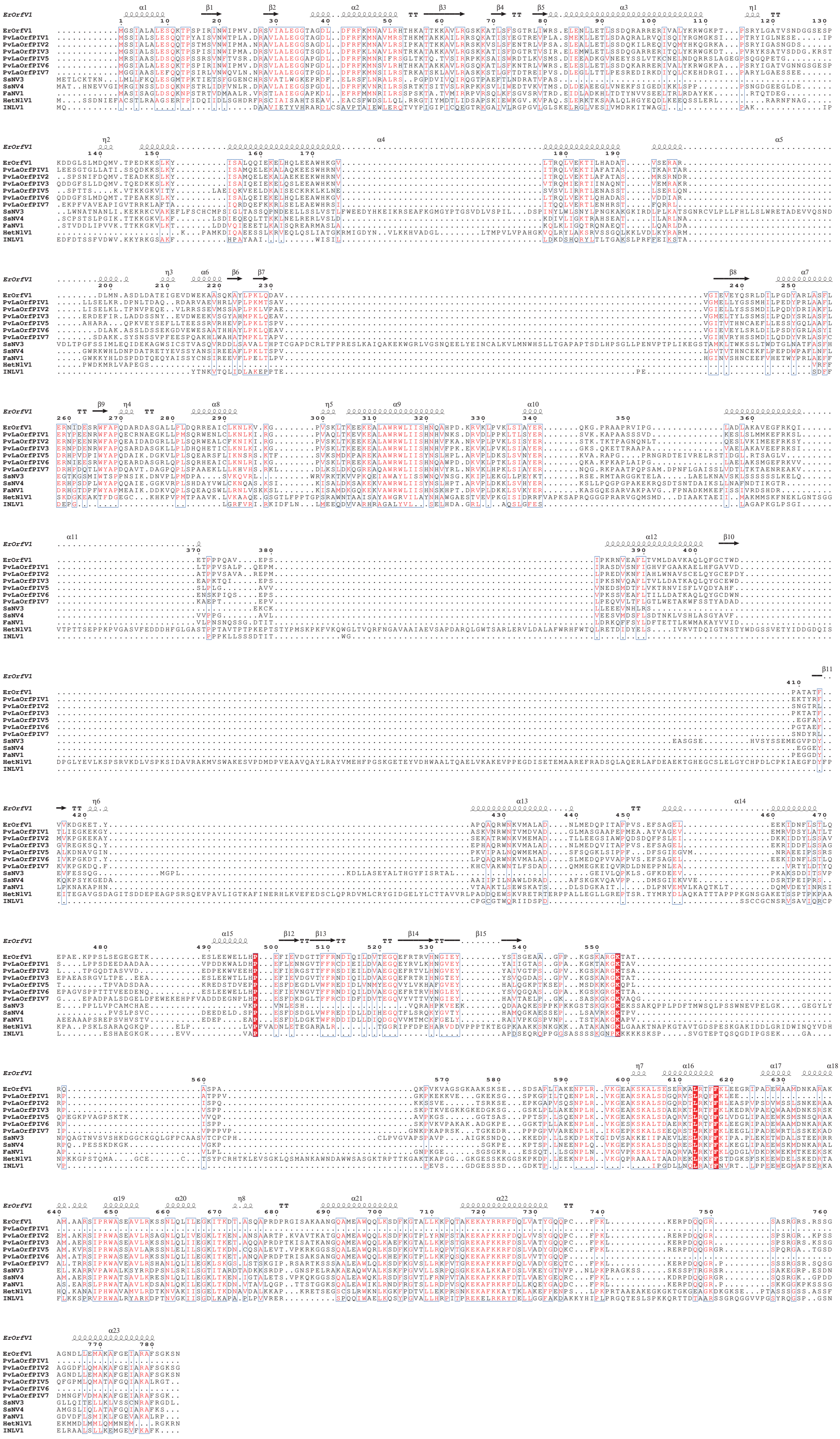
ErOrfV1
PvLaOrfPIV1
PvLaOrfPIV2
PvLaOrfPIV3
PvLaOrfPIV5
PvLaOrfPIV6
PvLaOrfPIV7
SsNV3
SsNV4
FaNV1
HetNlV1
INLV1
