# Supplementary material for: Cross-species transmission of a novel bisegmented orfanplasmovirus in the phytopathogenic fungus Exserohilum rostratum
Source: Front Microbiol. 2024 May 23;15:1409677. doi: 10.3389/fmicb.2024.1409677 (PMC11153860; doi:10.3389/fmicb.2024.1409677)
Supplement: Supplementary file 9 [file Image_4.PDF]

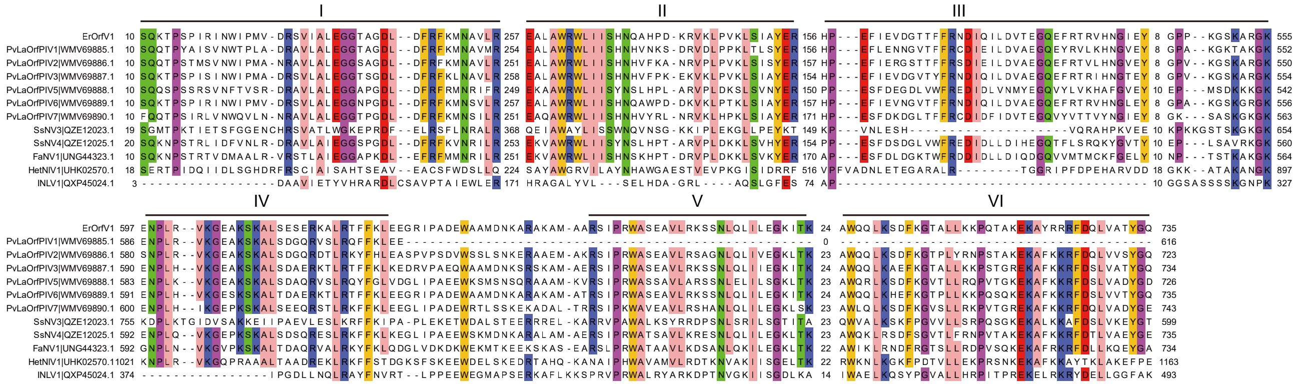
I
II
III
ErOrfV1 10 SQKTPSPIRINWIPMV-DRSVIALEGGTAGDL--DFRFKMNAVLR 257 EALAWRWLIISHNQAHPD-KRVKLPVKLSIAYER 156 HP---EFIEVDGTTFFRNDIQILDVTEGQEFRTRVHNGIEY 8 GPP--KGSKARGK 555
PvLaOrfPIV1|WMV69885.1 10 SQQTPYAISVNWTPLA-DRAVLALEGGTAGDL--DFRFKMNAVMR 254 EALAWRWLIISHNHVNKS-DRVDLPPKLTLSYER 156 HP---EFLENNGVTFFRCDIEILDVAEGQEYRVLKHNGVEY 8 GPA--KGKTAKGK 552
PvLaOrfPIV2|WMV69886.1 10 SQQTPSTMSVNWIPMA-NRAVLALEGGSAGDL--DFRFKMNAVLR 251 EALAWRWLIISHNHVFKA-NRVPLPVKLSISYER 157 HP---EFIERGSTTFFRSDIEILDVAEGQEFRTVLHNGVEY 8 GPV--KGSKARGK 550
PvLaOrfPIV3|WMV69887.1 10 SQKTPSPISINWIPMA-DRSVLALEGGTSGDL--DFRFKLNAVLR 258 EALAWRWLIISHNHVFPK-EKVKLPVKLSIAYER 154 HP---EFIEVDGVTYFRNDIQILDVAEGQEFRTRIHNGTEY 8 GPV--KGSKARGK 554
PvLaOrfPIV5|WMV69888.1 10 SQQSPSSRSVNFTVSR-DRAVLALEGGAPGDL--DFRFRMNRIFR 249 EKAAWRWLIISYNSLHPL-ARVPLDKSLSVNYER 150 PP---ESFDEGDLVWFREDIDLVNMYEGQVYLVKHAFGVEY 10 EP---MSDKKGK 542
PvLaOrfPIV6|WMV69889.1 10 SQKTPSPIRINWIPMV-DRSVLALEGGNPGDL--DFRFKMNSVLR 257 EALAWRWLIISHNQAWPD-DKVKLPTKLSIAYER 157 HP---EFIEVNGVTFFRNDIQILDVAEGQEFRTRVHNGLEY 8 GPA--KGSKGRGK 556
PvLaOrfPIV7|WMV69890.1 10 FQQTPSIRLVNWQVLN-NRAVLALEGGTPGDL--DFRFKMNSILR 251 EAQAWRWLIISHNHVYRL-NRVKLHPKLSIAYER 171 HP---ESFDKDGLTFFRCDIDFIDVTEGQVYVTTVYNGLEY 8 GK---SASKGKGK 563
SsNV3|QZE12023.1 19 SGMTPKTIETSFGGENCHRSVATLWGKEPRDF--ELRSFLNRALR 368 QEIAWAYLISSWNQVNSG-KKIPLEKGLLPEYKT 149 KP---VNLESH--------------------VQRAHPKVEE 10 KPKKGSTSKGKGK 654
SsNV4|QZE12025.1 20 SQKNPSTRLIDFVNLR-DRAVLAIEGGSPGDL--DFRFKVNRILR 253 EKVAWRWLIISYNSLHPT-ARVPLDKKLSVHYER 154 PP---ESFDSDGLVWFREDIDLLDIHEGQTFLSRQKYGVTY 10 EP---LSAVRKGK 560
FaNV1|UNG44323.1 10 SQKNPSTRTVDMAALR-VRSTLAIEGGAPKDL--EFRFMMNRILR 251 EKVAWRWLIISHNSKHPT-DRVPLDKKLSVKYER 170 AP---ESFDLDGKTWFRDDIDLLDIQDGQVVMTMCKFGELY 10 NP---TSTKAKGK 564
HetNIV1|UHK02570.1 18 SERTPIDQIIDLSGHDRFRSCIAISAHTSEAV--EACSFWDSLLQ 224 SAYAWGRVILAYNHAWGAESTVEVPKGISIDRRF 516 VPFVADNLETEGARALR--------TGGRIPFDPEHARVDD 18 GKK--ATAKANGK 897
INLV1|QXP45024.1 3 ----------------DAAVIETYVHRARDLCSAVPTAIEWLER 171 HRAGALYVL---SELHDA-GRL----AQSLGFES 74 AP-- 10 GGSASSSSKGNPK 327
IV
V
VI
ErOrfV1 597 ENPLR--VKGEAKSKALSESERKALRTFFKLEEGRIPADEWAAMDNKARAKAM-AARSIPRWASEAVLRKSSNLQLILEGKITK 24 AWQQLKSDFKGTALLKKPQTAKEKAYRRRFDQLVATYGQ 735
PvLaOrfPIV1|WMV69885.1 586 ENPLR--VKGEAKSKALSDGQRVSLRQFFKLEE-…- 0 -…- 616
PvLaOrfPIV2|WMV69886.1 580 SNPLR--VKGEAKSKALSDGQRDTLRKYFHLEASPVPSDVWSSLSNKERAAEM-AKRSIPRWASEAVLRSAGNLQLIVEGKLTK 23 AWQQLKSDFKGTPLYRNPSTAKEKAFKKRFDQLVVSYGQ 723
PvLaOrfPIV3|WMV69887.1 590 ENPLH--VKGEAKSKALSDAERKTLRTFFKLKEDRVPAEQWAAMDNKSRSKAM-AERSIPRWASEAVLRKSSNLQLIIEGKITK 24 AWQQLKAEFKGTALLKKPSTGREKAFKKRFDSLVATYGQ 734
PvLaOrfPIV5|WMV69888.1 583 ENPLK--VKGEAKSKALSDAQRVSLRQYFGLVDGLIPAEEWKSMSNSQRAAAM-KVRSIPRWASSAVLARSSNLELILSGKLTK 23 AWQRLKSDFKGVTLLRQPVTGKEKAFKKRFDSLVADYGD 726
PvLaOrfPIV6|WMV69889.1 591 ENPLH--VKGESKSKALTDAERKTLRTFFKLEEGRIPADEWAAMDNKARAKAM-ATRSIPRWASEAVLRKSSNLQLILEGKITK 24 AWQQLKHEFKGTPLLKKPQSQKEKAFKKRFDSLVVTYGQ 735
PvLaOrfPIV7|WMV69890.1 600 ENPLH--IKGEPKSKALSEEQRSTLRKFFKLEEGLIPADEWRTLSSKEKADAL-TRRSIPKWAVEAVLRSHANLQLILEGKLSK 23 AWLQLKSDFKGVSLLRKPVTGREKAFKKRFDQLVADYGE 743
SsNV3|QZE12023.1 755 KDPLKTGIDVSAKKEIIPAEVLESLKRFFKIPA-PLEKETWDALSTEERREL-KARRVPAWALKSYRRDPSNLSRILSGTITA 23 QWVALKSKFPGVVLLSRPQSGKEKALKKAFDSLVKEYGT 599
SsNV4|QZE12025.1 592 ENPLQ--VKGEPKSKALSDSQRESLRKFFKLKEGLIPAEEWSKMDNKARALAM-AERSIPRWATSAVLKRESNLQLILEGKLTK 23 AWQKLRSDFSGVTLFRNPVTAKEKAFKKRFDTLVAEYGE 735
FaNV1|UNG44323.1 592 GNPLN--VKGVPKSKALTDAQRVALRKYFKLQDGLVDKDKWEKMTKEEKSKAS-EARSLPRWATAAVLKDSANLQKILEGKLTK 22 AWIKLRNDFRGTSLLRDPVSQKEKAFRKRFDLLKQEYGA 734
HetNIV1|UHK02570.1 1021 KNPLR--VKGQPRAAALTAADREKLRKFFSTDGKSFSKEEWDELSKEDRTAHQ-KANAIPHWAVAMVLRDTKNVAKIISGELTK 22 RWKNLKGKFPDTVLLEKPRSNKEKAFKKAYTKLAKEFPE 1163
INLV1|QXP45024.1 374 ----------------IPGDLLNQLRAYFNVRT--LPPEEWEGMAPSERKAFLKKSPRVPRWALRYARKDPTNVGKIISGDLKA 14 IWAELKQSYPGVALLHRPITPREKELRKRYDELLGGFAK 493
