# Supplementary material for: Cross-species transmission of a novel bisegmented orfanplasmovirus in the phytopathogenic fungus Exserohilum rostratum
Source: Front Microbiol. 2024 May 23;15:1409677. doi: 10.3389/fmicb.2024.1409677 (PMC11153860; doi:10.3389/fmicb.2024.1409677)
Supplement: Supplementary file 11 [file Image_6.PDF]

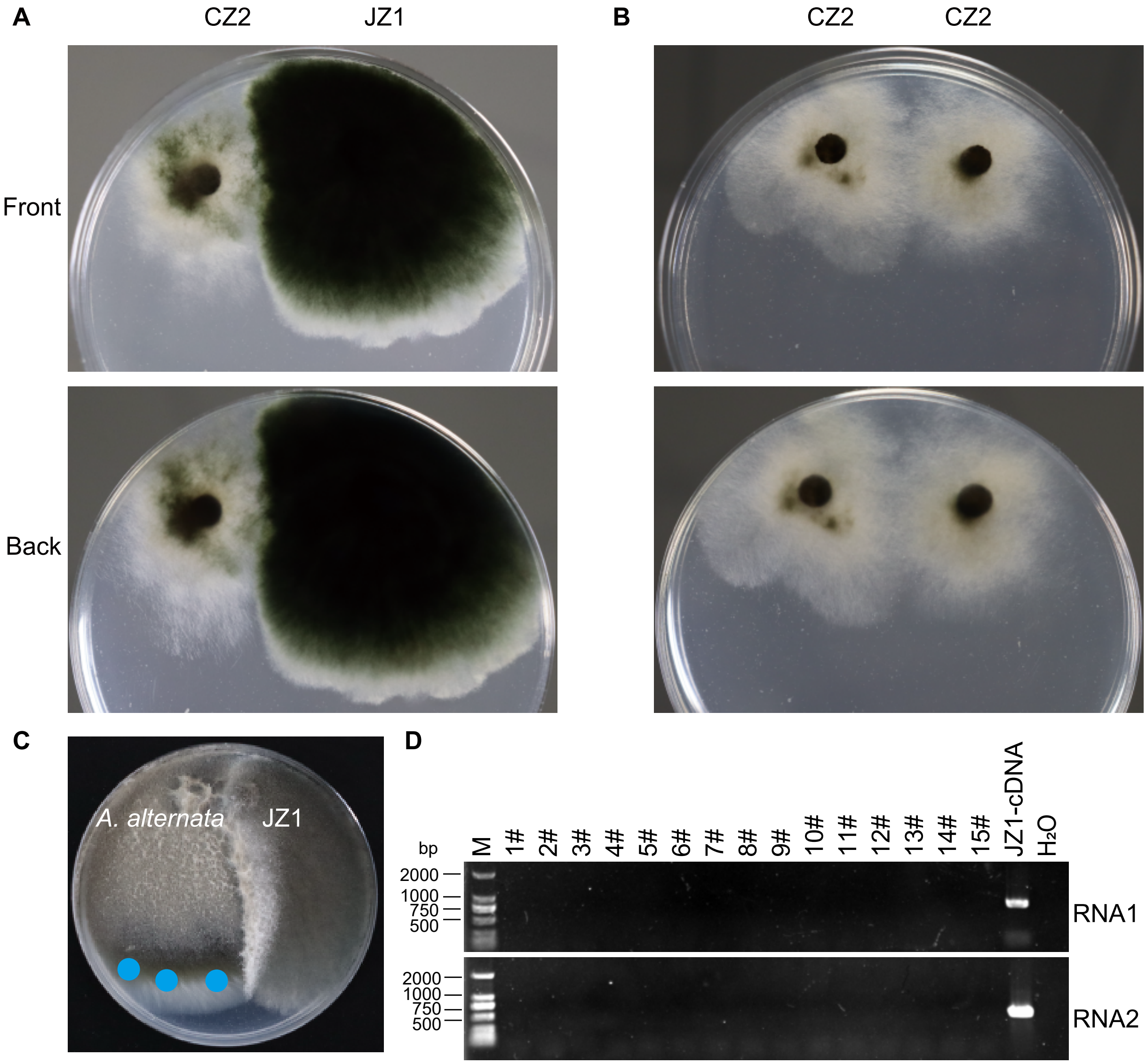

A
CZ2
JZ1
Front
Back
B
CZ2
CZ2
C
A. alternata
JZ1
D
bp
M
1#
2#
3#
4#
5#
6#
7#
8#
9#
10#
11#
12#
13#
14#
15#
JZ1-cDNA
H₂O
2000
1000
750
500
RNA1
2000
1000
750
500
RNA2
